# Morphological and genetic characterization of the muscadine fruit abscission zone

**DOI:** 10.1093/hr/uhae227

**Published:** 2024-08-09

**Authors:** Alana R Brinley, Patrick J Conner, Fahong Yu, Ali Sarkhosh, Tie Liu

**Affiliations:** Horticultural Sciences Department, University of Florida, Gainesville, FL 32611, USA; Department of Horticulture, University of Georgia, Tifton, GA 31793, USA; Bioinformatics, Interdisciplinary Center for Biotechnology Research, University of Florida, Gainesville, FL 32611, USA; Horticultural Sciences Department, University of Florida, Gainesville, FL 32611, USA; Horticultural Sciences Department, University of Florida, Gainesville, FL 32611, USA

## Abstract

Muscadines face limitations to fresh market production due to high manual labor costs. Mechanical harvesting holds promise for reducing the costs associated with muscadine production but requires cultivars with easily detached fruit at maturity. This study aimed to determine muscadine fruit and pedicel characteristics influencing fruit detachment force (FDF) and to unravel the genes, hormones, and regulatory networks governing muscadine abscission. We characterized the FDF of muscadine fruit across 18 genotypes and at four developmental stages. Following this, we performed a transcriptome analysis using the mature pedicel tissue of two genotypes, a genotype with high FDF at maturity and a genotype with low FDF at maturity, to identify differentially expressed and uniquely expressed genes contributing to fruit detachment. We found that pedicel length, pedicel–fruit junction area, and fruit diameter positively correlated with FDF. This study also identified novel candidate genes, transcription factor families, and pathways associated with muscadine fruit abscission. These findings provide valuable knowledge on the progression of fruit abscission and insights for reducing FDF, particularly in developing machine-harvestable muscadine cultivars and fostering sustainability and efficiency in muscadine production.

## Introduction

The muscadine (*Muscadinia rotundifolia* Michx.) is native to the southeastern USA and is the first grape cultivated in North America [1]. Muscadines are well known for their resistance to the pests and pathogens that commonly afflict bunch grape production as well as for their high nutritional content, which comprises antioxidants, dietary fibers, vitamin C, and vitamin K [2, 3]. Despite their attractive features, muscadine production remains confined to a regional market in the southeastern USA. Unlike the more common bunch grape (*Vitis vinifera* L.*),* which is thin-skinned and grown and harvested in bunches, muscadines are thick-skinned, grow in small clusters, and have fruit that abscise individually at a region between the stem (pedicel) and the fruit. Notably, muscadines exhibit uneven ripening and are non-climacteric, hence they must ripen on the vine prior to harvesting. These features require each muscadine to be individually hand-harvested at maturity before naturally abscising and becoming unsalable [4, 5].

The costs associated with hand-harvesting muscadine can consume between 30% and 40% of their gross production revenue [5]. Mechanical harvesting technology has shown promise in improving efficiency and reducing labor costs in grape production [6, 7]. Initial efforts in mechanical harvesting focused on understanding machine–plant interactions, particularly the transmission of vibrations and their impact on fruit yield and quality [7]. Modern grape harvesters employ advanced shaking and collection mechanisms designed to optimize fruit detachment while minimizing damage to both the vine and the fruit. However, adapting these technologies to ensure minimal fruit damage remains a challenge, especially for fresh market grape production where fruit integrity is crucial [6, 8]. Muscadines have the potential to be mechanically harvested for the fresh market. Having an abscission zone between the pedicel and fruit allows fruit to be harvested without their stem and their thick skin allows them to withstand high levels of mechanical stress without fruit damage.

To date, only one study has assessed muscadine cultivar suitability for mechanical harvest. Balerdi and Mortenson (1973) evaluated the fruit detachment force (FDF) of mature muscadine fruit from 24 cultivars, comparing FDF to fruit weight and providing a suitability rating for marketing [9]. FDF is a measure of the force required to remove an individual fruit from its pedicel. It is a valuable metric for determining a crop’s suitability for mechanical harvest [10]. However, this study is limited to mature fruit and does not assess fruit physical features that potentially impact FDF such as pedicel, fruit, and pedicel–fruit junction morphology. Furthermore, it does not consider the underlying genetic components that may influence fruit detachment behavior across genotypes, which are essential for muscadine breeders to develop superior cultivars for mechanical harvesting.

FDF is typically used to quantitatively measure abscission, the biological process that leads to the shedding of plant aerial organs. This process occurs at a specialized region of small cells with densely packed cytoplasm called the abscission zone (AZ) [11, 12]. Molecular studies in model plant species *Arabidopsis thaliana* and *Solanum lycopersicum* have identified several essential genes and hormones involved in AZ differentiation and separation. As a process, abscission can be delineated into four fundamental phases. In the first phase of abscission, the AZ differentiates at a region between two organs [13, 14]. In tomatoes, MADS-box transcription factors JOINTLESS and JOINTLESS2 control AZ differentiation. The *jointless* and *jointless2* mutants completely lack a pedicel abscission zone [12]. Two other MADS-box transcription factors, MACROCALYX (MC) and SlMBP21, regulate pedicel AZ development alongside JOINTLESS. In non-model species, *JOINTLESS* homologs *MdJa* and *MdJb* in apple (*Malus domestica*) and *PsJOINTLESS* in the Korla fragrant pear (*Pyrus sinkiangensis* Yü) were found to have functions similar to that of *JOINTLESS* in tomato, suggesting that the MADS-box family may have conserved functions in regulating AZ development [15–17].

In the second phase of abscission, the AZ is ready to perceive hormonal signals and activate cell separation [18]. Several phytohormones are known to play critical roles in abscission. Ethylene (ETH), abscisic acid (ABA), and methyl jasmonate (MeJA) tend to positively regulate abscission, whereas auxin (IAA), cytokinin (CTK), and gibberellin (GA) are negative regulators of abscission [13]. ETH is generally regarded as a vital hormone for abscission and was the first hormone identified in the abscission process [19, 20]. In tomatoes, reduced expression of the ETH receptor-encoding gene, *LeETR1*, results in delayed fruit abscission [21]. Recent studies assessing fruitlet abscission in litchi found essential ETH biosynthetic genes (*LcACO2*, *LcACO3*, *LcACS1*, *LcACS4,* and *LcACS7*) are upregulated in the AZ alongside ETH receptor-encoding genes *LcETR2* and *LcERFs* (Ethylene Response Factors) [22, 23]. A similar pattern was observed in apple fruitlets [24, 25].

In the final stages of abscission, hydrolytic enzymes dissolve the middle lamella between AZ cells, and the AZ scar’s final differentiation co-occurs [26, 27]. Several cell wall-degrading enzymes hydrolyze these cell walls, including cellulases, polygalacturonases, pectin methylesterases, and pectate lyases [28]. The AZ scar is a protective layer for the main plant body and the abscised organ, preventing pathogen entry by sealing off the region where the shedding organ was previously attached [13].

Identifying key morphological traits, genes, and pathways involved in abscission and discovering the molecular mechanism of abscission and fruit detachment are essential for improving crop yield and harvesting efficiency. For example, identifying *JOINTLESS* in tomato was revolutionary for machine harvesting [29]. Traditional tomatoes have an AZ or joint within their pedicel, causing them to retain a stem after harvest. This residual pedicel makes mechanical harvesting difficult, increasing the likelihood of mechanical damage. Plants with the *jointless* mutation fail to form functional AZs in their fruit pedicels, and therefore, they can be easily harvested without their stems, reducing mechanical damage and optimizing harvest operations [29]. Studying the molecular mechanisms associated with abscission can provide valuable insights into FDF determination, which is critical for mechanically harvesting fresh market fruits and vegetables. Despite this, the molecular mechanisms underlying AZ development and fruit detachment have yet to be thoroughly investigated in many non-model species, including muscadine.

In this study, we conducted a morphological and physical analysis of the development of the pedicel and pedicel–fruit junction across 18 muscadine genotypes. Additionally, we performed a transcriptome analysis using the pedicel tissue of two muscadine genotypes displaying opposite detachment phenotypes. By studying grape physical characteristics such as AZ morphology, fruit and pedicel characteristics, and FDF in conjunction with a transcriptomic analysis, we offer a comprehensive approach to studying muscadine abscission on a level that has not been previously reported in any grape species. This study identifies several muscadine genotypes with the potential for mechanical harvesting. Further, we discovered positive correlations between muscadine FDF and AZ area, pedicel length, and fruit diameter. Through genome-wide expression profiling, pathway enrichment, and Arabidopsis mutant analyses, we identified several possible candidate genes, transcription factor families, and biological pathways involved in muscadine abscission.

## Materials and methods

### Plant materials

Eighteen muscadine genotypes were grown at the University of Florida’s Plant Science Research and Education Unit in Citra, FL. Ten genotypes were developed by the University of Georgia’s muscadine breeding program in Tifton, GA (Ga. 8–1-12, Ga. 6–1-269, Ga. 6–6-358, Ga. 12–3-22, Ga. 10–1-329, Ga. 12–5-46, Ga. 10–1-294, Ga. 10–1-222, Ga. 13–4-79, and Ga. 13–4-2) (See Table S1 for their berry characteristics). The remaining eight were released cultivars (‘Supreme’, ‘Paulk’, ‘Hall’, ‘Granny Val’, ‘Alachua’, ‘Noble’, ‘Triumph’, and ‘Carlos’). Fruit were harvested at four fruit developmental stages (pea-size, bunch closure, veraison, and maturity) [30].

### Fruit detachment force

FDF was measured using an IMADA DS2–4 force gauge (IMADA Inc., Northbrook, IL, USA). Fruit of similar size and color were harvested in the morning with their pedicels attached and brought back to the lab on ice; FDF was assessed on the same day as harvest. Individual fruits were detached using the handheld force probe by securing the pedicel with forceps and pulling the probe uniformly away from the pedicel. The maximum force required for fruit removal was recorded. FDF was assessed using fruit from all 18 genotypes at each developmental stage. Replicates of five at each developmental stage were used for analysis. A photograph of the system used can be found in Fig. S1.

### Abscission zone measurements and visualization

Photos of the pedicel–fruit junction, pedicel tissue, and whole fruit at each developmental stage were taken using a Nikon DX camera (AF-S NIKKOR 18–55 mm 1:3.5–5.6G) and a Leica light microscope (LEICA DMC4500, Wetzlar, Germany). ImageJ (Version 1.51) was then used to measure the pedicel–fruit junction area, pedicel length, and fruit diameter for all genotypes using five replicates each [31].

### Scanning electron microscopy

The pedicel-fruit fracture planes of two muscadine genotypes (Ga. 12–3-22 and Ga. 6–1-269) with two replicates each were examined using scanning electron microscopy (SEM). Tissues were harvested and stored at −20°C until further processing. Stem scar regions were dissected from the samples and stored in Trump’s fixative at 4°C for 16 h. Samples were fixed in 2% osmium tetroxide buffered in PBS and dehydrated using a graded ethanol series from 25% to 100%. Samples were transferred to a Tousimis Autosamdri-815 CPD (Tousimis Research Corporation, Rockville, MD, USA) using bone-dry CO_2_. Specimens were mounted on Cambridge-style SEM stubs using 12-mm carbon tabs and colloidal graphite, then coated with gold–palladium using a Desk V sputter coater (Denton DeskV, Moorestown, NJ, USA) with argon gas. Then, all samples were examined via secondary electrons (SE) at a high vacuum, 5 kV, on an FE-SEM (SU-5000; Hitachi High Technologies America, Schaumburg, IL, USA).

### RNA extraction, library preparation, and sequencing

Pedicel tissue from genotypes Ga. 12–3-22 and Ga. 6–1-269 were collected at two developmental stages (pea-size and maturity). The total RNA was isolated using the Nucleospin RNA Plant and Fungi Mini-kit (Macherey-Nagel, PA, USA). The RNA was treated with DNase using the PureLink™ DNase Set (Invitrogen™, CA, USA) and then subjected to a lithium chloride (LiCl) precipitation step, as described by Reid et al. (2006) [32]. RNA-seq libraries were constructed at the ICBR Gene Expression and Genotyping Core Lab using the NEBNext® Ultra™ Directional RNA Library Prep Kit for Illumina (New England Biolabs, Ipswich, MA, USA). The RNA integrity numbers (RIN) >5.6 were used. Sequencing was performed on an Illumina NovaSeq 6000-S4 (2 × 150 cycles) at ICBR (RRID: SCR_019145). Each sample had three biological replicates.

### RNA data processing and analysis

Reads were trimmed with the Cutadapt (v3.4) program to remove adaptors and low-quality bases with a phred-like score of < 20 and reads < 65 bases [33]. The genome of *Muscadine rotundifolia* ‘Noble’ from Park et al. (2022) was used as the reference sequences for RNA-seq analysis [34]. The cleaned reads were individually mapped to the reference genome using the STAR package (Spliced Transcripts Alignment to a Reference, v2.7.9a) [35]. Mapping results were processed with the HTSeq (High-Throughput Sequence Analysis in Python, v0.11.2) [36], samtools, and scripts developed in-house at ICBR of UF to remove potential polymerase chain reaction (PCR) duplicates and count uniquely mapped reads for gene expression analysis. The R-package DESeq-2 was used to identify significantly upregulated and downregulated genes within genotypes [37]. The log_2_ fold change (log_2_FC) of counted reads in mature pedicel tissue using the pea-size stage as a control was calculated. A *p*-value of 0.05 and a log_2_FC of ≥ 2 were used to determine differentially expressed genes (DEGs). Gene ontology (GO-term) analysis and pathway prediction were performed using ShinyGo (Version 0.741) [38]. Transcription factor analysis was performed using the Plant Transcription Factor Database (PlantRegMap/PlantTFDB v5.0) [39]. Electronic fluorescent pictographs (eFP) were generated using the Bio-Analytic Resource (BAR) at the University of Toronto online ‘Grape eFP Browser’ [40].

### Real-time quantitative PCR analysis

To validate the expression of select candidate genes, real-time quantitative PCR (RT-qPCR) analysis using two technical replicates from each of the three biological replicates were used. The cDNA was synthesized using the iScript™ cDNA synthesis kit from Bio-Rad (Hercules, CA, USA). The grape actin gene (*VvActin*) was used as an internal reference control. The ΔΔCT technique was used to determine relative gene expression.

### Arabidopsis mutant analysis

The closest Arabidopsis homologs of selected grape candidate genes were identified using NCBI BLAST. T-DNA insertion mutant seeds were obtained from the Arabidopsis Biological Research Center (ABRC, Columbus, Ohio, USA). All seeds were homozygous for the TDNA insert. Arabidopsis wild-type (Col-0) and mutant seeds were sterilized with 70% ethanol for 2 min, followed by 7 min in 50% bleach, then washed five times with sterile ddH_2_O. Seeds were plated on a solid MS medium containing basal salts (PhytoTech, Lenexa, KS, USA), 0.05% MES, and 0.05% sucrose (pH 5.7). Seedlings were grown on MS medium for 14 days under long-day conditions (16 h light / 8 h dark) at 25°C, then transplanted to a potting medium, where they continued to grow under the same conditions in a growth chamber. Photographs of floral organs and siliques were taken after forming the first 10 siliques, following floral organ positioning based on anthesis as position 1 [41, 42]. Images of siliques were captured using a Leica microscope and camera.

### Statistical analysis

Linear regression analysis was performed using Microsoft Excel for Windows (Version 2310s). Statistical analysis and visualization of the FDF data across genotype and developmental stage were performed using Microsoft Excel for Windows (Version 2310) and Python (Version 3.8) using the following libraries: Pandas (Version 1.3.3) for data manipulation and analysis [43] and Matplotlib (Version 3.4.3) and Seaborn (Version 0.11.2) for generating graphs and visualizations [44, 45].

## Results

### Muscadine fruit detachment force varies throughout development and across genotypes

**Figure 1:**
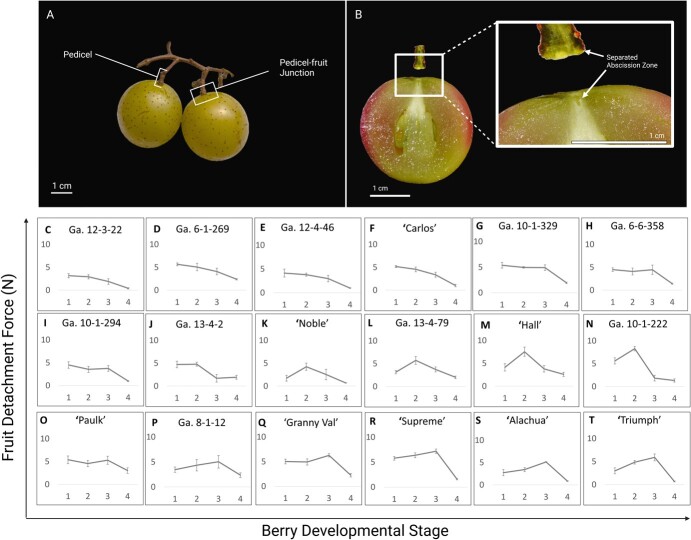
Morphology of muscadine pedicel, pedicel–fruit junction (A), and AZ (B). FDF (N) assessment across genotype throughout fruit development. The X-axis represents fruit developmental stage as denoted by 1: pea size, 2: bunch closure, 3: veraison, and 4: maturity. The Y-axis is the mean FDF in Newtons. (Error bars represent SE) (*N* = 5). Graphs show data for all genotypes: (C) Ga. 12–3-22, (D) Ga. 6–1-269, (E) Ga. 12–5-46, (F) ‘Carlos’, (G) Ga. 10–1-329, (H) Ga. 6–6-358, (I) Ga. 10–1-294, (J) Ga. 13–4-2, (K) ‘Noble’, (L) Ga. 13–4-79, (M) ‘Hall’, (N) Ga. 10–1-222, (O) ‘Paulk’, (P) GA. 8–1-12, (Q) ‘Granny Val’, (R) ‘Supreme’, (S) ‘Alachua’, (T) ‘Triumph’. Figure created with BioRender.com.

To better understand how the tensile strength of the AZ changes throughout development, we assessed FDF at four developmental stages (pea-size, bunch closure, veraison, and maturity) (Fig. S2) and across 18 genotypes. We found that FDF varies widely across genotype and developmental stage; however, we identified four discernable patterns. In 8 of the 18 genotypes, FDF decreased with the progression of development, where the highest FDF was observed at the pea-size stage (Fig. 1C–J). Four of these eight genotypes showed a gradual decrease in FDF throughout development (Fig. 1C–F). The remaining four saw gradual decreases in FDF throughout the first three developmental stages and a substantial drop in FDF between veraison and maturity (Fig. 1G–J). A third distinct pattern was observed in the following four genotypes, which saw an increase in FDF from pea-size to bunch closure, followed by a gradual decrease in FDF to maturity (Fig. 1K–N). The six remaining genotypes experienced the highest FDF at veraison, typically followed by a stark decrease in FDF at maturity (Fig. 1O–T). Despite different FDF patterns throughout development, most of the genotypes experienced the steepest decline in FDF between veraison and maturity, suggesting that the process of cell separation is initiated and completed during this time.

To observe physical changes in AZ integrity, we took photos of the pedicel–fruit junction throughout development and across the 18 genotypes (Fig. S3, Fig. S4). Additionally, we split the genotypes into one of three categories depending on their mean FDF at maturity (Stage 4): ‘Weak Attachment’ (FDF < 1 N), ‘Moderate Attachment’ (1 N < FDF < 2 N), and ‘Strong Attachment’ (FDF > 2 N). Splitting the genotypes into categories allowed us to identify patterns related to pedicel–fruit junction morphology and fruit detachment phenotype. The six ‘Weak Attachment’ genotypes included Ga. 12–3-22, ‘Triumph’, ‘Noble’, ‘Alachua’, Ga. 12–5-46, and Ga. 10–1-294. We identified six additional genotypes in the ‘Moderate Attachment’ category: ‘Carlos’, Ga. 10–1-222, Ga. 6–6-358, ‘Supreme’, Ga. 13–4-2, and Ga. 10–1-329. The final six genotypes were placed in the ‘Strong Attachment’ category: Ga. 13–4-79, ‘Granny Val’ Ga. 8–1-12, Ga. 6–1-269, ‘Hall’, and ‘Paulk’.

Microscopic examination of the pedicel–fruit junctions across the three categorized genotypes revealed an association between FDF and tissue breakdown at this junction. Genotypes with lower FDFs exhibited more pronounced tissue breakdown at the pedicel–fruit junction, whereas those with higher FDFs showed less tissue degradation. Precisely, Fig. S4A–C displays the pedicel–fruit junctions of mature fruit in genotypes Ga. 12–3-22, ‘Alachua’, and Ga. 12–5-46 belonging to the ‘Weak Attachment’ category, showcasing a higher degree of tissue degradation compared to genotypes classified under ‘Moderate Attachment’ (Fig. S4D–F) and ‘Strong Attachment’ Fig. S4G–I). The variability in tissue degradation is particularly evident in the degree of vascular bundle maintenance (an example of an intact vascular bundle is indicated by the arrow in Fig. S4I). While there are no vascular bundles maintained in the three ‘Weak Attachment’ genotypes shown in Fig. S4A–C, the genotypes Ga. 6–6-358 and ‘Supreme’ in the ‘Moderate Attachment’ category exhibited partial vascular bundle maintenance (Fig. S4E–F). The last three genotypes in the ‘Strong Attachment’ category, ‘Granny Val’, Ga. 6–1-269, and ‘Paulk’, had the highest degree of vascular bundle maintenance, with the two genotypes with the highest FDFs (Ga. 6–1-269 (mean FDF = 2.37 N) and ‘Paulk’ (mean FDF = 3.03 N)), maintaining two to three clear bundles at maturity (Fig. S4G–I). There were no evident patterns in tissue breakdown and maintenance across the categories throughout development (Fig. S3). This may be because the pictures were too macroscopic to observe the changes underlying differences in fruit detachment phenotypes at these early stages. The pedicel-fruit junctions for all genotypes throughout development are shown in Fig. S3. The patterns observed at maturity in the nine genotypes shown in Fig. S4 are consistent throughout all 18 genotypes. These results suggest that tissue breakdown at and around vascular bundles at the pedicel–fruit junction may contribute to differences in FDF at maturity.

**Figure 2:**
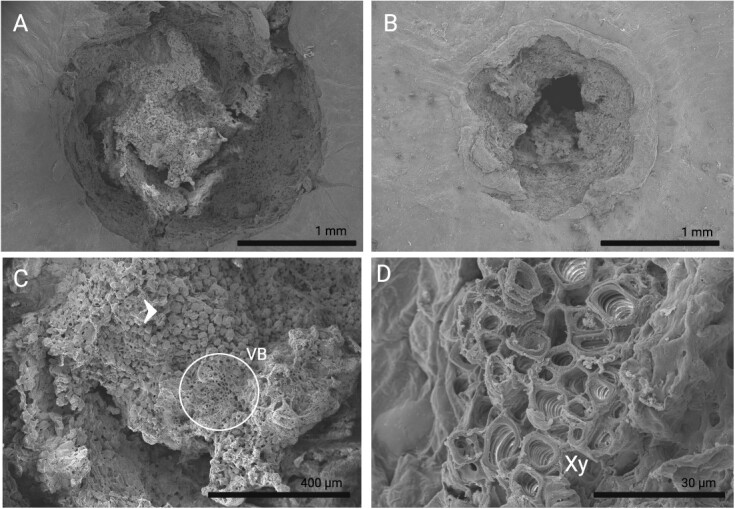
Scanning electron micrographs of the pedicel-fruit fracture plane in (A) Ga. 6–1-269 (S), and (B) Ga. 12–3-22 (W). (C) Close-up of the Ga. 6–1-269 (S) pedicel-fruit fracture plane showing small, circular cells that make up the AZ (indicated by the arrow). Vascular bundle (VB) regions are shown within the circle. (D) Xylem tissue (Xy) in the Ga. 6–1-269 (S) pedicel-fruit fracture plane.

To further investigate the morphological differences in AZ, SEM was performed on two genotypes with opposing FDFs. The pedicel–fruit fracture planes of mature fruit from Ga. 6–1-269 (S, strongly attached), which exhibits a high FDF, and Ga. 12–3-22 (W, weakly attached), which has a low FDF, were photographed and compared (Fig. 2A–B). Both genotypes exhibit small, rounded cells throughout the fracture plane, as indicated by the arrow in Fig. 2C. One notable difference between the genotypes is the composition of the fracture plane’s center. Ga. 6–1-269 (S) has tissue build-up and protrusion at its center, while Ga. 12–3-22 (W) displays a pit at the center of the fracture plane. More connective tissue in Ga. 6–1-269 (S) may contribute to its higher FDF compared to Ga. 12–3-22 (W), which lacks this connective tissue. Additionally, the larger tissue area in Ga. 6–1-269 (S) correlates with a greater presence of vascular bundle tissue. The circle in Fig. 2C highlights the vascular bundles within the fracture plane, which contain the lignified xylem (Fig. 2D). These findings emphasize the importance of vascular bundle maintenance and tissue integrity in influencing FDF and abscission behavior in muscadine.

To assess the influence of fruit and pedicel characteristics of muscadine on FDF, we measured the area of the pedicel–fruit junction, fruit diameter, and pedicel length of mature muscadine fruit (Table S2). A diagram demonstrating how each muscadine characteristic (pedicel–fruit junction area, fruit diameter, and pedicel length) was measured can be found in Fig. S5. With these measurements, we performed a linear regression analysis to determine the effect of each fruit characteristic previously mentioned on FDF (Fig. S6). Means for each measured parameter (pedicel–fruit junction area, fruit diameter, and pedicel length) were correlated with mean FDF across genotypes. All measurements were taken using only mature fruit. A significant positive correlation between all three characteristics and FDF was observed. There was a moderate positive correlation between the pedicel–fruit junction area and FDF, with a correlation coefficient of R = 0.534. Similarly, we observed a moderate positive correlation between pedicel length and FDF, with a correlation coefficient of R = 0.632. A strong correlation was observed between fruit diameter and FDF with a correlation coefficient of R = 0.831. Therefore, fruit with larger AZs, longer pedicels, and larger fruit size were more likely to have higher FDFs and hence, be more difficult to remove from their pedicels.

### Differentially expressed genes were identified across the strong and weak FDF genotypes

**Figure 3:**
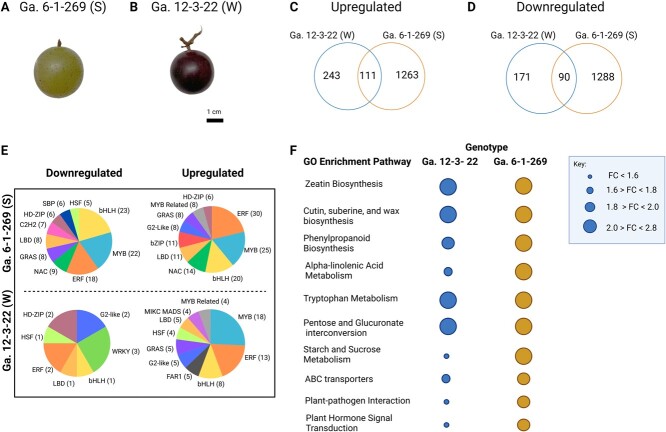
(A) Mature fruit from the Ga. 6–1-269 (Strong, S) genotype. (B) Mature fruit from the Ga. 12–3-22 (Weak, W) genotype. (C) Number of upregulated DEGS from Stage 1 to Stage 4 independently and simultaneously expressed by Ga. 6–1-269 (S) and Ga. 12–3-22 (W). (D) Number of downregulated DEGS from Stage 1 to Stage 4 independently and simultaneously expressed by Ga. 6–1-269 (S) and Ga. 12–3-22 (W). (E) Pie charts displaying the proportion of genes encoding transcription factors upregulated and downregulated in Ga. 12–3-22 (W) and Ga. 6–1-269 (S). The top 10 transcription factor families in each category are shown apart from the downregulated genes encoding TFs in Ga. 12–3-22 (W), where only seven families were identified. The different colors are representative of different transcription factor families. (F) Gene ontology enrichment diagram of the top enriched pathways in Ga. 12–3-22 (W) and Ga. 6–1-269 (S). Only DEGS with a log_2_FC ≥ 1 were analyzed, FDR ≤ 0.05. Figure created with BioRender.com.

To identify genes and molecular pathways contributing to the variation in fruit detachment phenotype and abscission behavior in muscadine, we performed RNA sequencing analysis on the pedicel tissue of two select genotypes identified through our fruit detachment surveys (Fig. 1). These genotypes were previously used for SEM and were selected as benchmarks for a genotype with fruit that detach easily from its pedicel at maturity versus a genotype with fruit firmly attached to their pedicel at maturity. Ga. 6–1-269 (Strong attachment, S). A large bronze grape (Fig. 3A) was selected as the benchmark for strong attachment due to its high FDF at maturity (FDF = 2.37 ± 0.31) (Table S2). The Ga. 12–3-22 (Weak attachment, W) genotype, a medium-large red grape (Fig. 3B), was selected as the easily abscised benchmark due to its low FDF at maturity (FDF = 0.41 ± 0.30) (Table S2). While Ga. 6–1-269 (S) did not exhibit the highest FDF across the genotypes used, it was selected because it follows an FDF pattern similar to Ga. 12–3-22 (W) throughout development. This is shown in Fig. 1C–D, where both genotypes experience their highest FDF at Stage 1, which gradually declines throughout development. Therefore, we compared gene expression across these genotypes based on gene expression changes from Stage 1, when fruit are pea-size (stage with the highest FDF), to Stage 4, at maturity (stage with lowest FDF). The gene transcripts with significant expression levels (*p* ≤ 0.05) and log_2_FC ≥ 1 were used to summarize the RNA sequencing data, gene ontology enrichment analysis, and transcription factor analysis.

Among genes that were upregulated in the pedicel tissue from Stage 1 to Stage 4, we identified 243 genes independently expressed in Ga. 12–3-22 (W) and 1263 genes independently expressed in Ga. 6–1-269 (S) (Fig. 3C). There were 111 genes commonly upregulated from Stage 1 to Stage 4 (Fig. 3C). Among the genes downregulated in the pedicel tissue from Stage 1 to Stage 4 were 171 genes independently expressed in Ga. 12–3-22 (W) and 1288 genes independently expressed in Ga. 6–1-269 (S). There were 90 genes commonly downregulated from Stage 1 to Stage 4 (Fig. 3D). Genes that were commonly upregulated and commonly downregulated across both genotypes are shown in Table S3.

### The bHLH, MYB, and ERF transcription factor families were differentially expressed during muscadine abscission

A closer look at the top 10 representative transcription factor (TF) families that are encoded by differentially expressed genes from the pea-size stage to maturity across genotypes reveals the major involvement of several different TF families (Fig. 3E). Among DEGs that encode TFs, the bHLH, MYB, and ERF families are the most abundant cross both genotypes. In Ga. 6–1-269 (S), there are 23 downregulated genes encoding TFs in the bHLH family, 22 members from MYB, and 18 members from ERF. Some of the most significantly downregulated genes encoding TFs from these families in Ga. 6–1-269 (S) include *VIT_01S0026G00010* (*VvbHLH168*), *VIT_17S0000G03550* (*VvbHLH93*), *VIT_02S0025G00320* (*VvMYB86*), *VIT_12S0134G00570* (*VvMYB39*), *VIT_11S0016G05340* (*VvWIN1*, an ERF member), and *VIT_13S0047G00340* (*VvWRI1*, an ERF member). We identified genes encoding 30 ERF members, 25 MYB members, and 20 bHLH members upregulated in Ga. 6–1-269 (S) including *VIT_14S0081G00730* (*VvERF.C3*), *VIT_17S0000G04480* (*VvRAP2.11*), *VIT_00S0203G00170* (*VvMYB4*), *VIT_08S0007G06310* (*VvPHL5*), *VIT_14S0081G00720* (*VvbHLH111*), and *VIT_01S0010G02070* (*VvbHLH162*).

There were significantly fewer total downregulated genes encoding TFs in Ga. 12–3-22 (W) when compared to Ga. 6–1-269 (S), which aligns with the RNA-seq data shown in Fig. 3C–D. In Ga. 12–3-22 (W), there is one member in the bHLH family and two members of the ERF family downregulated; they are *VIT_07S0191G00240* (*VvbHLH94*), *VIT_18S0001G10150* (*VvERF020*), and *VIT_02S0025G04440* (*VvERF026*), respectively. Similar to Ga. 6–1-269 (S), ERF, MYB, and bHLH were highly represented among upregulated genes encoding TFs in Ga. 12–3-22 (W) with 18 MYB members, 23 ERF members, and 8 bHLH members being upregulated from pea-size stage to maturity. These include *VIT_01S0011G03730* (*VvMYB62*), *VIT_15S0048G02120* (*MYB*), and *VIT_01S0244G00010* (*VvbHLH104*). All genes encoding TFs identified through the TF analysis can be found in Table S4. Genes encoding TFs in the ERF, MYB, and bHLH families that were significantly upregulated or downregulated (*p* ≤ 0.05 and log_2_FC ≥ 2) across both genotypes are shown in Table S5. Some additional families that were commonly in the top represented TF families across upregulated and downregulated DEGs in both genotypes include HD-ZIP, GRAS, and LBD. Notably, the G2-like and the Myb-Related families were only highly represented across upregulated DEGs.

### Gene ontology enrichment analysis reveals several key pathways in muscadine abscission

To further elucidate the pathways involved in FDF determination and abscission, we performed a gene ontology enrichment analysis using significantly upregulated and downregulated genes (*p* ≤ 0.05, log_2_FC ≥ 1) from the pea-size stage to maturity in both genotypes. Figure 3F shows the top 10 enriched pathways shared by both genotypes and their relative enrichment based on the RNA-seq results (FDR ≤ 0.05). Several pathways share high enrichment across genotypes, including ‘zeatin biosynthesis’, ‘cutin, suberin, and wax biosynthesis’, and ‘tryptophan metabolism, pentose, and glucuronate interconversion’, which all have an enrichment FC between 2.0 and 2.8 in both genotypes. Ga. 6–1-269 (S) shows greater enrichment than Ga. 12–3-22 (W) in several pathways, including ‘phenylpropanoid biosynthesis’, ‘alpha-linolenic acid metabolism’, ‘starch and sucrose metabolism’, ‘ABC transporters', ‘plant and pathogen interaction’, and ‘plant and hormone signal transduction’ pathways (Fig. 3F).

### Muscadine candidate genes are differentially expressed within the fruit AZ

To gain insight into genes contributing to the differences in FDF observed in Ga. 12–3-22 (W) and Ga. 6–1-269 (S), we selected genes with opposite expression patterns in the pedicel tissue of Ga. 12–3-22 (W) and Ga. 6–1-269 (S) from pea-size to maturity. The opposite differential expression of genes can indicate opposing biological processes or regulatory mechanisms. For example, genes upregulated in Ga. 12–3-22 (W) while downregulated in Ga. 6–1-269 (S) can highlight potential biomarkers for weak FDF.

We identified 36 genes that were significantly oppositely regulated across genotype (log_2_FC ≥ 1, *p* ≤ 0.05), shown in Table 1. Of these 36 genes, 24 were upregulated in Ga. 12–3-22 (W) and downregulated in Ga. 6–1-269 (S). Among these genes was *VIT_18S0001G12900* (‘jasmonate O-methyltransferase’, *VvJMT*), a gene involved in jasmonic acid (JA) biosynthesis. This gene was also downregulated during shade-induced flower abscission in *V. vinifera* [46]. The gene *VIT_10S0003G05420* (‘berberine bridge enzyme’, *VvBBE-LIKE15*) was also upregulated in Ga. 12–3-22 (W) and downregulated in Ga. 6–1-269 (S) and is involved in lignin biosynthesis, a pathway with high involvement in abscission [47, 48]. Additionally, we identified 12 genes that were downregulated in Ga. 12–3-22 (W) and upregulated in Ga. 6–1-269 (S). Among these genes was *VIT_07S0129G00330* (‘LOB domain-containing protein 38’, *VvLBD38*) a gene in the Lateral Organ Boundaries (LOB) family. LOB family genes exhibit expression patterns similar to Arabidopsis *HAESA*, a leucine-rich repeat receptor kinase required for floral organ abscission [49, 50]. Similarly, *VIT_12S0035G02090* (‘probable leucine-rich repeat receptor-like protein kinase’) is upregulated in Ga. 12–3-22 (W) and downregulated in Ga. 6–1-269 (S) and is homologous to Arabidopsis *HPCA1* (*AT5G49760*). *HPCA1* is a gene involved in hydrogen peroxide (H_2_O_2_) perception, and H_2_O_2_ has vital roles in lignin polymerization and abscission [48, 51].

**Table 1:** Genes that display opposite regulation across Ga. 12–3-22 (W) and Ga. 6–1-269 (S) with a log_2_FC ≥ 1 and a *p*-value ≤ 0.05

**Ensembl gene ID**	**Putative ATH orthologue**	**NCBI protein ID**	**Ga. 6–1-269 (S)** f**old change (log**_**2**_**)**	**Ga. 12–3-22 (W)** f**old change (log**_**2**_**)**	**Gene annotation**
*Upregulated in Ga. 12–3-22 and downregulated in Ga. 6–1-269*					
*VIT_18S0001G12900*	*AT1G19640.1*	XP_002281579.1	−4.12	2.02	jasmonate O-methyltransferase
*VIT_00S0372G00070*	*AT1G61680.1*	NP_001267895.1	−4.03	2.11	(3S,6E)-nerolidol synthase / Nerolidol synthase
*VIT_10S0003G05420*	*AT2G34790.1*	XP_002268361.1	−3.95	2.14	berberine bridge enzyme-like 15
*VIT_09S0002G05810*	*AT1G15460.1*	XP_010654729.1	−3.83	2.76	boron transporter 4
*VIT_11S0016G04920*	*AT1G52820.1*	XP_002285052.1	−3.71	8.29	early nodulin-93-like
*VIT_14S0068G01760*	*AT3G01980.3*	XP_002271350.1	−3.18	1.51	levodione reductase
*VIT_00S0266G00010*	*AT1G61680.1*	NP_001268060.1	−2.98	1.55	(3S,6E)-nerolidol synthase / Nerolidol synthase
*VIT_02S0012G00530*	*AT2G35390.2*	XP_002282232.1	−2.82	1.03	ribose-phosphate pyrophosphokinase 1 isoform X1
*VIT_02S0025G01940*	*AT4G23990.1*	XP_002264341.2	−2.76	2.32	cellulose synthase-like protein G3
*VIT_04S0008G04920*	*AT1G52820.1*	XP_002284931.1	−2.74	1.58	probable 2-oxoglutarate-dependent dioxygenase AOP1
*VIT_15S0021G00330*	*AT1G32450.1*	XP_002265725.3	−2.45	3.43	protein NRT1/ PTR FAMILY 7.3
*VIT_15S0048G01590*	*AT2G45550.1*	XP_002276094.1	−2.18	1.48	cytochrome P450 76 T24-like
*VIT_05S0049G02240*	*AT3G11480.1*	XP_002285517.1	−1.88	2.14	membrane protein PM19L
*VIT_14S0060G02340*	*AT5G28540.1*	NP_001290009.1	−1.83	3.04	heat shock 70 kDa protein BIP1-like
*VIT_02S0025G02560*	*AT1G64660.1*	XP_002280162.1	−1.74	1.30	methionine gamma-lyase
*VIT_16S0100G00290*	*AT5G51970.1*	XP_010662490.1	−1.70	1.50	L-idonate 5-dehydrogenase isoform X1
*VIT_02S0154G00480*	*AT4G25200.1*	XP_002270632.1	−1.61	2.10	“small heat shock protein
*VIT_00S0779G00010*	*AT4G38060.3*	XP_003635478.1	−1.59	1.25	Uncharacterized
*VIT_08S0007G01030*	*AT2G37770.2*	XP_002272909.1	−1.45	1.99	“NADPH-dependent aldo-keto reductase
*VIT_04S0023G02240*	*AT1G04560.1*	XP_002263018.1	−1.43	1.28	salicylate carboxymethyltransferase-like isoform X2
*VIT_08S0105G00430*	*AT5G05580.1*	XP_002264350.1	−1.31	1.41	“omega-3 fatty acid desaturase
*VIT_13S0019G02820*	*AT1G53540.1*	XP_002281285.1	−1.28	1.90	18.2 kDa class I heat shock protein
*VIT_11S0016G05010*	*AT1G80160.3*	XP_002285087.1	−1.26	2.13	metallothiol transferase FosB-like
*VIT_07S0151G00800*	*AT3G22550.1*	XP_003632307.1	−1.08	1.57	protein MARD1-like
*Downregulated in Ga. 12–3-22 and upregulated in Ga. 6–1-269*					
*VIT_07S0129G00330*	*AT4G37540.1*	XP_002284296.1	1.03	−1.30	LOB domain-containing protein 38
*VIT_08S0007G01050*	*AT2G37770.2*	XP_010654238.1	1.12	−1.53	“NADPH-dependent aldo-keto reductase
*VIT_18S0157G00180*	*AT4G39460.2*	XP_010665070.1	1.12	−2.60	“S-adenosylmethionine carrier 1
*VIT_17S0000G01110*	*AT5G50740.3*	XP_002284075.1	1.34	−2.91	heavy metal-associated isoprenylated plant protein 7
*VIT_12S0035G02090*	*AT5G49760.1*	XP_019079160.1	1.42	−1.54	probable leucine-rich repeat receptor-like protein kinase
*VIT_12S0028G03270*	*AT5G44210.1*	XP_002276215.1	1.47	−1.68	ethylene-responsive transcription factor 4
*VIT_01S0011G03110*	*AT1G25550.1*	XP_003631224.1	1.85	−1.97	myb family transcription factor EFM
*VIT_05S0049G00930*	*AT5G43360.1*	XP_002281264.1	2.07	−1.71	probable inorganic phosphate transporter 1–3
*VIT_00S0317G00110*	*AT2G39420.1*	XP_002282295.1	2.19	−1.30	caffeoylshikimate esterase isoform X1
*VIT_01S0026G00880*	*AT5G44210.1*	XP_003631291.1	2.80	−1.72	protein JINGUBANG
*VIT_01S0011G03400*	*AT1G68570.1*	XP_010647749.1	2.95	−1.33	protein NRT1/ PTR FAMILY 3.1
*VIT_01S0026G01490*	*AT1G69870.1*	XP_002269340.1	9.72	−4.09	protein NRT1/ PTR FAMILY 2.13

To validate the RNA-seq results, we performed RT-qPCR using 20 genes identified from the RNA-seq dataset. These 20 genes fall into one of three categories. The first 10 genes are oppositely regulated across Ga. 12–3-22 (W) and Ga. 6–1-269 (S) and were selected from the 36 genes listed in Table 1. The second category consists of five genes uniquely upregulated in Ga. 6–1-269 (S) but not significantly expressed in Ga. 12–3-22 (W). The final category consists of five genes uniquely upregulated in Ga. 12–3-22 (W) but not significantly expressed in Ga. 6–1-269 (S). In addition to the criteria for each category, genes were selected based on their high log_2_FC differences across genotypes or because they belong to pathways and/or gene families with previously reported involvement in abscission. The selected genes in their respective categories and corresponding primers are shown in Table S6. Following RT-qPCR, we calculated the log_2_FC of the transcripts obtained from the pea-size stage to maturity and compared it to the log_2_FC obtained from RNA-seq. We found that the log_2_FC from the RT-qPCR data resembled that of the RNA-seq data (Fig. S7).

To further validate that these genes exhibited expression localized to AZ regions, we used the eFPs generated by the University of Toronto’s Bio-Analytic Resource for Plant Biology online database (BAR.utoronto.ca). This resource uses publicly available expression data to temporally predict tissue-specific expression. Here we used the 20 genes previously identified for RT-qPCR validation to examine tissue-specific expression of the genes in *V. vinifera*. Pictographs showing the tissue-specific expression of these 20 genes are shown in Figs S8 , S9, and S10. We found that the expression of the 20 genes studied thus far were predominantly localized to plant regions with abscission or dehiscence zones, such as the rachis, floral organs, siliques, and leaves (Fig. S8, S9, S10).

### The *cad9* loss-of-function mutant displays delayed floral organ abscission

**Figure 4:**
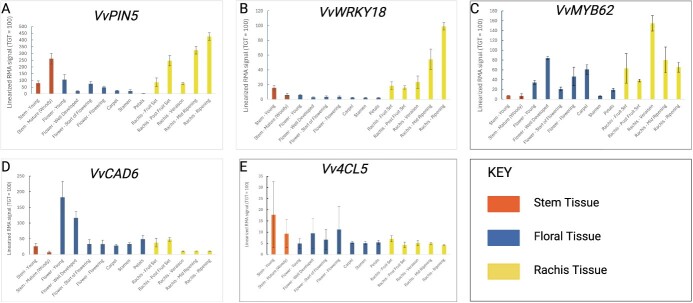
Linearized temporal and tissue-specific (stem, flower, and rachis) expression values based on data collected by Fasoli et al. [52] for (A) *VIT_04S0023G00320* (*VvPIN5*), (B) *VIT_04S0008G05750* (*VvWRKY18*), (C) *VIT_01S0011G03730* (*VvMYB62*), (D) *VIT_18S0001G14910* (*VvCAD6*), and (E) *VIT_13S0019G05260* (*Vv4CL5*). Figure created with BioRender.com.

To functionally validate select genes for their role in abscission, we conducted a loss-of-function mutant analysis in Arabidopsis. From the 20 grape genes previously analyzed using RT-qPCR and eFP analysis, five genes were selected based on their predicted expression localization to AZ-containing tissues, such as the rachis, floral tissue, or stem. The predicted tissue-specific expression values of these five genes are shown in Fig. 4. These genes include one hormone-related gene *VIT_04S0023G00320* (‘auxin efflux carrier component 5’, *VvPIN5*), a plant and pathogen interaction gene, *VIT_04S0008G05750* (‘WRKY18-like’ or *VvWRKY18*), and gene encoding a MYB-Family TF *VIT_01S0011G03730* (‘MYB62-like’, *VvMYB62*). These genes all display expression localization to the grape rachis (Fig. 4A–C). The last two genes are involved in the phenylpropanoid biosynthesis process, *VIT_18S0001G14910* (‘probable cinnamyl alcohol dehydrogenase 6’, *VvCAD6*) and *VIT_13S0019G05260* (‘4-coumarate–CoA ligase-like 5’, *Vv4CL5*). These genes are localized to the floral organs and stem tissues, respectively (Fig. 4D–E). Further, these genes were selected because the loss-of-function mutant seeds of their orthologues in *A. thaliana* were available through the Arabidopsis Biological Research Center (ABRC, Columbus, OH). To perform the loss-of-function mutant analysis, the closest Arabidopsis orthologue of each gene above was determined using NCBI BLAST and Arabidopsis mutants generated by T-DNA insertion were ordered through the ABRC. All seeds ordered were homozygous for the insertion to ensure the stable integration and inheritance of the transgene. Table S7 contains all information about the Arabidopsis genes targeted in this mutant analysis, including their *Vitis* homologue, ABRC identifier, and expression across Ga. 12–3-22 (W) and Ga. 6–1-269 (S).

**Figure 5:**
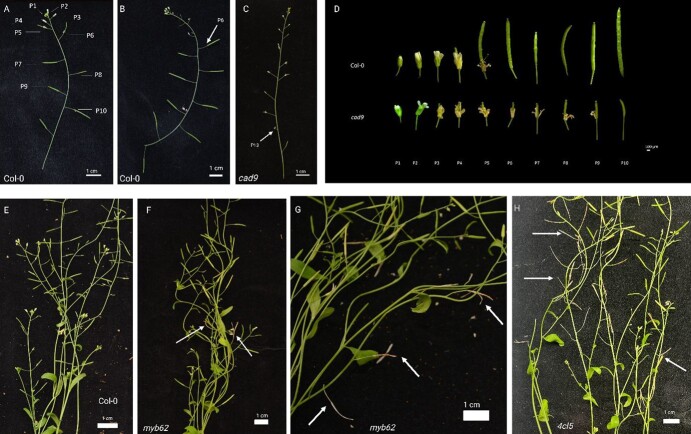
Functional validation of candidate genes in *A. thaliana* by loss-of-function mutation. (A) Floral organ positioning in Col-0 wild-type plant. (B) Floral organ positioning in the wild-type Col-0. The arrow indicates P6 where floral organs start to abscise. (C) Floral organ and silique positioning on *cad9.* The arrow indicates P13, the last position that retains its floral organs. (D) Microscope images of siliques and floral organ abscission behavior across floral position in wild-type (Col-0) and loss-of-function mutant *cad9*. (E) Wild-type Col-0 plant, 6 weeks after germination. (F) *myb62* mutant 6 weeks after germination. The arrows point to senesced siliques. (G) Senesced siliques on *myb62* mutant. (H) *4cl5* mutant 6 weeks after germination. The arrows point to senesced siliques. Figure created with BioRender.com.

Flower and silique development in Arabidopsis follow a distinct pattern that can be denoted with position numbers [53]. Figure 5A shows where these positions are located, with positions 1 through 10 denoted by ‘P1’ through ‘P10’. In this system, the youngest organs are located at P1, and the position number increases with increasing maturity. Arabidopsis do not typically abscise their leaves or siliques, but they do abscise their floral organs (petals, sepals, and anthers) [54]. Therefore, to determine if the selected mutants experienced abscission deficient or accelerated abscission phenotypes, we measured the time required for each mutant to shed its floral organs according to floral and silique positioning. When compared to the Columbia (Col-0) wild-type plants, four of the five genotypes abscised their floral organs similar to the control group at position 5 or 6 after 6 weeks of growth (Fig. 5B). Plants with mutations to the *CINNAMYL ALCOHOL DEHYDROGENASE 9* (*CAD9*) gene were the only mutants with significant defects in floral organ abscission. The *CAD9* gene is the closest Arabidopsis homolog of *VIT_18S0001G14910* (‘probable cinnamyl alcohol dehydrogenase 6’, *VvCAD6*), a gene that displayed high upregulation in the weakly attached genotype, Ga. 12–3-22 (log_2_FC = 7.7) but was not significantly upregulated or downregulated in Ga. 6–1-269. The *cad9* mutants maintained their floral organs until siliques were between position 9 and position 13 (Fig. 5C). The *cad9* mutants also show reduced silique development, where siliques fail to elongate and exhibit reduced seed development (Fig. 5D).

In the Arabidopsis with mutations to *MYB62 (AT1G68320)*, the orthologue of *VIT_01S0011G03730* (*VvMYB62*), and *4CL5 (AT1G20480)*, the orthologue of *VIT_13S0019G05260* (*Vv4CL5*), we observed accelerated senescence of silique tissue and accelerated seed dehiscence when compared to the Col-0 wild type, suggesting these genes might play distinct roles in grape AZ development (Fig. 5A–H). The plants with mutations to *WRKY18* (*AT2G25000*), the orthologue of *VIT_04S0008G05750* (*VvWRKY18*), and *PIN5* (*AT5G16530*), the orthologue of *VIT_04S0023G00320* (*VvPIN5*), did not display noticeable abscission defective or accelerated phenotypes.

## Discussion

Muscadine production has faced limitations in market reach due to the high costs associated with manual harvesting. The potential solution lies in implementing mechanical harvesting, a method dependent on fruit that easily detach from their pedicels. To date, only one study has assessed muscadine cultivar suitability for mechanical harvest. The study by Balerdi and Mortenson (1973) assessed muscadine FDF of mature fruit from 24 cultivars. However, their study only compared FDF of mature fruit to fruit weight and a suitability rating for marketing [9]. Our study uniquely assessed the FDF of muscadines across four developmental stages and drew comparisons between FDF and fruit and pedicel characteristics. Further, this is the first study to examine muscadine abscission at the molecular level.

We found that FDF varied widely across developmental stage and across genotype. FDF tended to peak early in development and was consistently lowest at maturity. Similar results were observed in a 2016 study looking at FDF of sour cherry, where it was found that FDF gradually decreased throughout development, similar to the genotypes seen in Fig. 1C–F [55]. Another study looking at FDF throughout development in Tabasco pepper observed a similar FDF pattern to the genotypes in Fig. 1K–T, where FDF is highest in the middle stages of development [56].

As a quantitative tool for measuring abscission, we expected FDF to closely follow the stages of abscission. Since AZ dissolution is the last stage of abscission, it was expected that fruit would have the lowest FDF at maturity. However, the vast differences observed in peak FDF across genotype were unexpected. There are a limited number of studies looking at FDF throughout development and across multiple genotypes in any horticultural crop, so there is no well-accepted explanation for this. However, the differences observed in tissue breakdown at the pedicel–fruit junction (Fig. S3) suggest that the rate of AZ breakdown differs widely across genotype. We observed that genotypes with lower FDF at maturity experienced a higher degree of tissue breakdown between the fruit and the pedicel, likely contributing to their low FDF (Fig. S3). Fruit with high FDF exhibited less tissue breakdown and maintained their vascular bundles, hence maintaining the heavily lignified xylem at maturity. This was further observed using SEM (Fig. 2C–D). Lignin is a tensile biopolymer that enhances plant cell wall rigidity [57]. Maintaining these vascular bundles at the pedicel–fruit junction is likely a key contributor to higher FDFs in these genotypes. Further, it is possible that larger fruit tend to have higher FDFs because of more extensive vascular networks.

Notably, these results provide promise for the deployment of mechanical harvest in muscadine even though they typically exhibit uneven ripening and require multiple harvests [58]. Large differentials between FDF in young and mature fruit can facilitate multiple harvests more easily by selectively harvesting mature fruit. Fruit that experience large differences in FDF between veraison and maturity, such as ‘Granny Val’, ‘Supreme’, ‘Alachua’, and ‘Triumph’ (Fig. 1Q–T), are good candidates for machine harvesting when looking to optimize yields through multiple selective harvests.

At the molecular level, differential gene expression analysis showed significant enrichment of genes involved in zeatin biosynthesis and cutin, suberin, and wax biosynthesis across both genotypes (Fig. 3F). Zeatin is a type of cytokinin, which is a large family of signaling molecules necessary for regulating plant growth and development [59]. Zeatin is one of the most active and abundant cytokinins present in plants; therefore, its significant enrichment across genotypes could be related to plant growth and development rather than abscission. However, previous research suggests that cytokinins play a role in the negative regulation of abscission [13]. The common enrichment of this pathway might suggest a common involvement of cytokinins in abscission. Similarly, the high enrichment of cutin, suberin, and wax biosynthesis genes (Fig. 3F) is likely due to the natural accumulation of wax on the fruit and pedicel surface throughout development [60]. However, the relationships between these pathways in the muscadine abscission process must be investigated further.

Other pathways showing differences in enrichment include alpha-linolenic acid metabolism, plant and pathogen interaction, and plant hormone signal transduction. Alpha-linolenic acid is an unsaturated fatty acid involved in the biosynthesis of plant antioxidants and JA [61]. One gene in this pathway, *VIT_18S0001G12900* (‘jasmonate O-methyltransferase’, *VvJMT*), displays significant downregulation in Ga. 6–1-269 (S) but is upregulated in Ga. 12–3-22 (W). A study by Fidelibus et al. (2022) reveals that exogenous JA application rapidly induces grape fruit abscission [62]. Upregulation of jasmonate O-methyltransferase in Ga. 12–3-22 (W) may result in increased JA biosynthesis, which likely induces the expression of genes encoding other signaling molecules such as ETH or cell wall-degrading enzymes.

TF analysis identified several key transcription factor families with possible roles as regulators of abscission in grape. Among the families identified, bHLH, ERF, and the MYB TF families were the most highly represented. The bHLH TFs make up a large group of TFs that regulate numerous developmental processes and stress responses [63]. In several cases, bHLH TFs regulate stress responses through the activation of JA and ETH signaling pathways [64, 65]. It is possible that the bHLH TFs have a dual role in stress response and abscission activation through the mediation of JA and ETH signaling. The bHLH transcription factor, MebHLH18, was shown to play a role in cassava leaf abscission during low-temperature stress. Increased expression of *MebHLH18* resulted in increased peroxidase (POD) activity, thus decreasing reactive oxygen species (ROS) activity and leaf abscission rates [66]. The higher abundance of bHLH TFs showing significant upregulation and downregulation across genotypes suggest that bHLH TFs play regulatory roles during abscission, possibly through altering ROS levels in the AZ or through hormone transduction.

Additionally, in both genotypes we see a greater number of genes encoding ERF TFs upregulated at the time of maturity, revealing a key role of ETH in muscadine fruit abscission in the later stages of development. Interestingly, *VvERF4* (*VIT_212S0028G03270*), is one of the only genes encoding an ERF TF displaying opposite regulation across the selected genotypes, with upregulated expression in Ga. 6–1-269 (S) and downregulated expression in Ga. 12–3-22 (W) (Table 1). While the involvement of *ERF4* in abscission has yet to be characterized, in watermelon, it was shown that *ClERF4* is a causative gene responsible for rind hardness [67]. Liao et al. (2020) found that *ClERF4* is a member of the Group IIId ERFs. The Arabidopsis members in Group IIId ERFs include *AtERF038*, *AtERF039*, *AtERF034*, and *AtERF035,* which were shown to be positive transcriptional regulators of genes related to primary cell wall biosynthesis [67, 68]. Upregulation of this gene in the strongly attached genotype may contribute to the increased expression of cell wall-modulating enzymes, including those involved in lignin and cellulose biosynthesis, thus establishing a stronger connection at the pedicel–fruit junction and contributing to higher FDFs at maturity. Currently, high doses of ethephon can induce the abscission of mature grapes within 1–2 weeks of treatment. However, exceeding 1000 ppm are needed, which leaves ethephon residues on grapes. This is already a concern for regulatory agencies, so identifying and targeting key ERF genes during grape breeding could benefit the grape industry [62].

**Figure 6:**
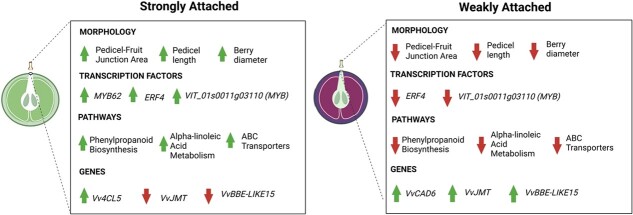
Schematic diagram of key findings, including morphological features, TFs, pathways, and candidate genes predicted to influence abscission and fruit detachment. Results are split into two categories: strongly attached genotype (left) and weakly attached genotype (right). Arrows indicate increase (upright arrow) or decrease (downward facing arrow) in size for morphology, upregulation (upright arrow) or downregulation (downward facing arrow) for transcription factors and genes, and higher (upright arrow) versus lower (downward facing arrow) enrichment for pathways. Figure created with BioRender.com.

Following the RNA-seq analysis, *VIT_18s0001g14910* (‘Cinnamyl alcohol dehydrogenase 6’, *VvCAD6*) was identified as a possible candidate gene in the abscission process based on its differential gene regulation across Ga. 12–3-22 (W) and Ga. 6–1-269 (S). Arabidopsis with mutations to *CAD9*, the closest Arabidopsis homolog of *VvCAD6*, exhibited defective floral organ abscission, strongly suggesting its role in the abscission process (Fig. 5C–D). *CAD9* is a member of the cinnamyl alcohol dehydrogenase family, which typically catalyzes the last steps of monolignol biosynthesis. The role of lignin in the abscission process is debated. This is likely because the cellular processes that occur proximal (fruit side) to the AZ are well understood, while the processes occurring in distal regions (pedicel side) are not [69]. Since the RNA-seq analysis performed in this study used pedicel tissue (distal side of the AZ) it offered a unique perspective of abscission, allowing us to identify the *CAD9* gene and other genes related to lignin biosynthesis that are exclusively expressed in the distal regions of the AZ. Lignin deposition typically occurs in these distal regions and is thought to be involved in forming the AZ scar at the separation site that protects the plant from pathogen attacks following abscission [13]. Merelo et al. (2017) suggested that lignin deposition in citrus may form a fracture layer that aids abscission [28]. However, Lee et al. (2018) found that in Arabidopsis floral organs, lignin acts as a brace that restricts the diffusion of cell wall-degrading enzymes to specific sites within the proximal side (floral side) of the AZ [48]. Therefore, a higher concentration of hydrolytic enzymes facilitates organ separation in these regions. In Arabidopsis mutants with disrupted lignin biosynthesis pathways, the failure to produce lignin results in the uncontrolled diffusion of cell wall-degrading enzymes, leading to delayed and irregular floral organ abscission.

In this study, we found that *VvCAD6* was significantly upregulated in the weakly attached cultivar, but significant expression was not detected in the strongly attached cultivar. The upregulation of *VvCAD6* in the weakly attached cultivar may contribute to forming a lignin fracture layer that aids abscission. Further, its upregulation may contribute to the formation of a lignin brace that directs cell wall-degrading enzymes to separation sites within this AZ, thus resulting in more efficient abscission. Recently, Li et al. (2023) identified *CAD9* as a negative regulator of *OVULE ACTIVATING FACTOR* (*OAF*) during ovule development in Arabidopsis [70]. However, the exact mechanisms of *CAD9* regulation were not characterized in detail. It is possible that *CAD9* similarly functions upstream of genes associated with Arabidopsis floral organ abscission.

*4CL5* is a gene in the 4-Coumarate:CoA ligase (4CL) family, which is also involved in the lignin biosynthesis process by encoding enzymes that catalyze the formation of CoA esters [71]. While there is limited research associated with *4CL5*, its orthologue, *VIT_13S0019G05260* (*Vv4CL5*), was highly upregulated in Ga. 6–1-269 (S) and was not significantly detected in the weakly attached genotype, Ga. 12–3-22. Therefore, the accelerated silique senescence and seed dehiscence observed in the *4CL5* mutant and high upregulation of *VIT_13S0019G05260* in the strongly attached genotype strongly suggest that this gene has a role in the negative regulation of abscission.

Our Arabidopsis mutant analysis also revealed that mutations to *MYB62* (*AT1G68320*), the Arabidopsis orthologue of *VIT_01S0011G03730* (*VvMYB62*), resulted in accelerated silique senescence and seed dehiscence compared to wild-type plants. In Arabidopsis, the genetic mechanisms regulating fruit (silique) and seed dehiscence are highly conserved [72]. Therefore, *MYB62* and *4CL5* may be involved in genetic networks that commonly regulate seed dehiscence and fruit abscission since we initially detected their expression in the fruit pedicel. *MYB62* is a gene encoding a MYB family TF that is widely known for its role in regulating GA biosynthesis in response to environmental stress [73]. Notably, a study by Liao et al. (2016) found that *MeMYB62* was highly expressed during ethylene-induced abscission of cassava leaf, suggesting its potential role in the abscission process [74]. Therefore, *VvMYB62*, which was highly upregulated in Ga. 12–3-22 (W) (log_2_FC = 8.46) but not significantly detected in Ga. 6–1-269 (S), may be similarly involved in muscadine abscission.

Our results provide valuable insights into the progression of abscission in muscadine; however, several of the study’s limitations need to be acknowledged. While the mature fruit (Stage 4) showed low variability in FDF across replicates in each genotype, the earliest stages (Stage 1 and 2) showed moderate variability across replicates, and veraison (Stage 3) showed the highest variability (Fig. S11). Veraison is the onset of ripening, and the dramatic changes occurring in the fruit’s internal environment likely influence the FDF; hence, prospective studies may consider increasing their sample sizes at variable growth stages and assessing maturity standards other than grape size and color, such as total soluble solids (TSS), fruit firmness, and the maturity index (TSS/titratable acidity (TA) ratio) in conjunction with FDF. Since abscission is a complex and stage-specific process, understanding how differing degrees of fruit maturity coincide with AZ separation is vital for determining the optimal harvesting time.

Moreover, mechanical harvesting exerts significant physical stress on the pedicel–fruit junction, increasing the likelihood of skin splitting and tearing in this region. Such mechanical damage renders the fruit unusable by promoting decay through entry points for pests and pathogens and enabling water loss [75]. However, this damage can be mitigated through the development of a dry stem scar, which forms when an abscission layer remains on the fruit after detachment from the pedicel [76, 77].

This study did not evaluate the physical development of stem scars across the genotypes assessed. However, dry scar development is essential for the successful marketing of fresh market muscadine. Future research should investigate the potential for stem scar development in genotypes with low FDF before implementing mechanical harvesting.

## Conclusion

This study looks deeply at the physical and molecular alterations occurring throughout development at the pedicel–fruit junction of muscadine. The FDF study demonstrates that the abscission process and AZ development likely occur at different rates in a genotype and developmental stage-dependent manner. This study also identifies physical features of muscadine fruit and pedicels that likely contribute to muscadine detachment force, including pedicel–fruit junction area, pedicel length, and fruit diameter that can be targets of muscadine breeding programs. Further, we offer several candidate genes, pathways, and TF families that can be the targets of future research related to abscission. Notably, this study reveals a key role of lignin biosynthesis and degradation in the pedicel-fruit connection. However, the role of lignin in abscission needs to be studied further due to its apparent complex roles interplaying between fruit and pedicel attachment versus detachment. Here we identify *VIT_18s0001g14910* (*VvCAD6*), *VIT_01S0011G03730* (*VvMYB62*), and *VIT_13S0019G05260* (*Vv4CL5*) for their possible roles in the muscadine abscission process. Future studies can assess the dynamics of *VvCAD6*, *Vv4CL5*, and lignin deposition in relation to abscission behavior. A summary of the key findings identified through this research is shown in Fig. 6.

This research represents a significant step forward in understanding fruit abscission, providing valuable insights that could have far-reaching implications for the cultivation and commercialization of muscadines and potentially other fruit crops. Future studies focusing on the detailed functional roles of identified genes and pathways and exploring the practical applications of this knowledge in various agricultural settings will be essential in fully harnessing the potential of these findings.

## Supplementary Material

Web_Material_uhae227

## Data Availability

The RNA-sequencing data in this paper has been deposited in the public expression repositories GEO (http://www.ncbi.nlm.nih.gov/geo, GSE259406, NCBI tracking system #24529064).
