# Supplementary material for: Morphological and genetic characterization of the muscadine fruit abscission zone
Source: Hortic Res. 2024 Aug 9;11(10):uhae227. doi: 10.1093/hr/uhae227 (PMC11480701; doi:10.1093/hr/uhae227)
Supplement: Web_Material_uhae227 [file web_material_uhae227.docx]

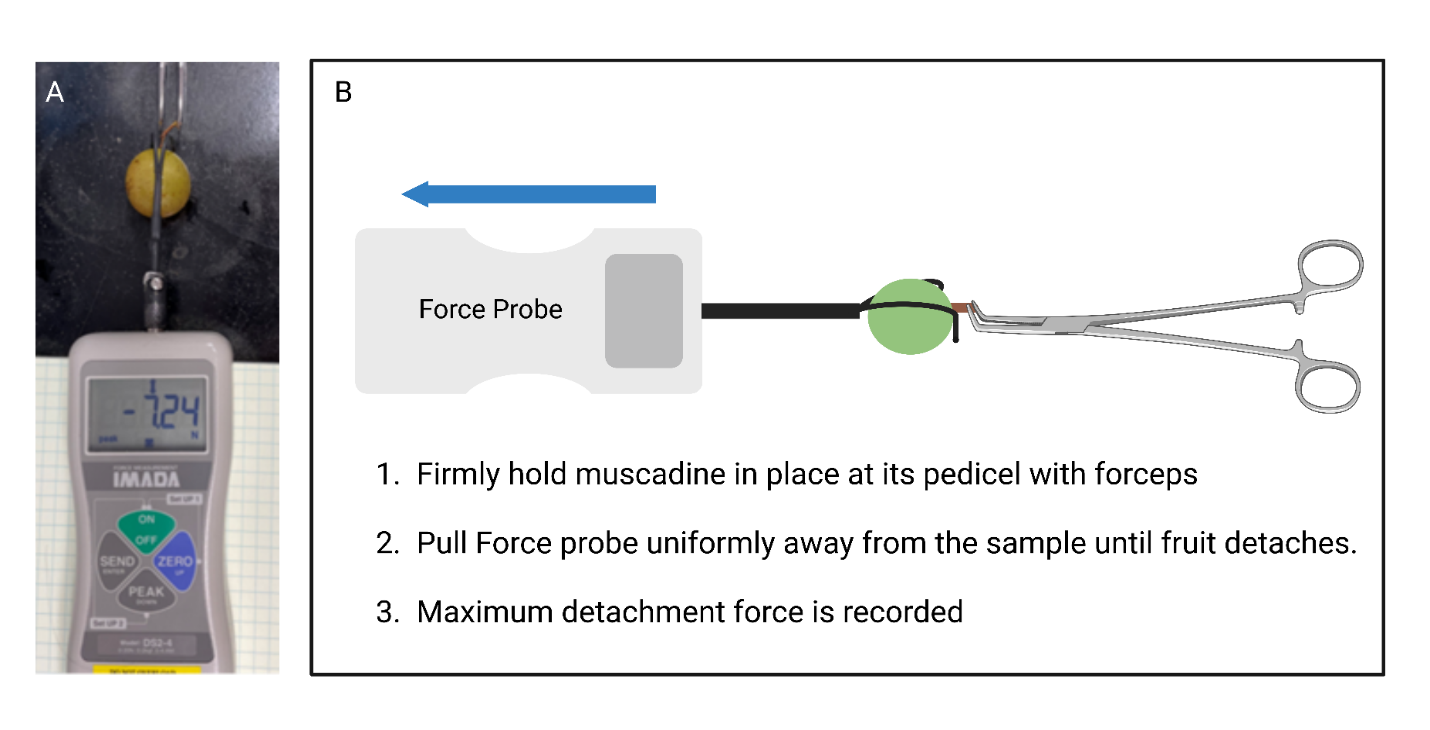


**Figure S1.** (A) IMADA force probe used in FDF experiments with proper set-up. (B) Diagram demonstrating technique for determining FDF.


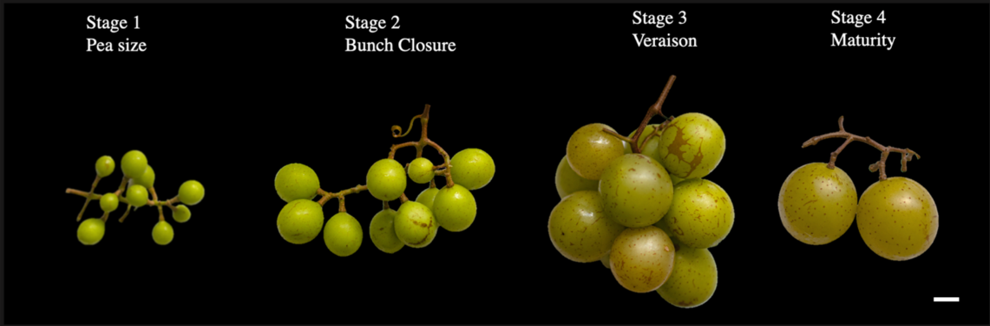


**Figure S2**. Muscadine berry developmental stages derived from Keller (2010). The scale bar is 1 cm.


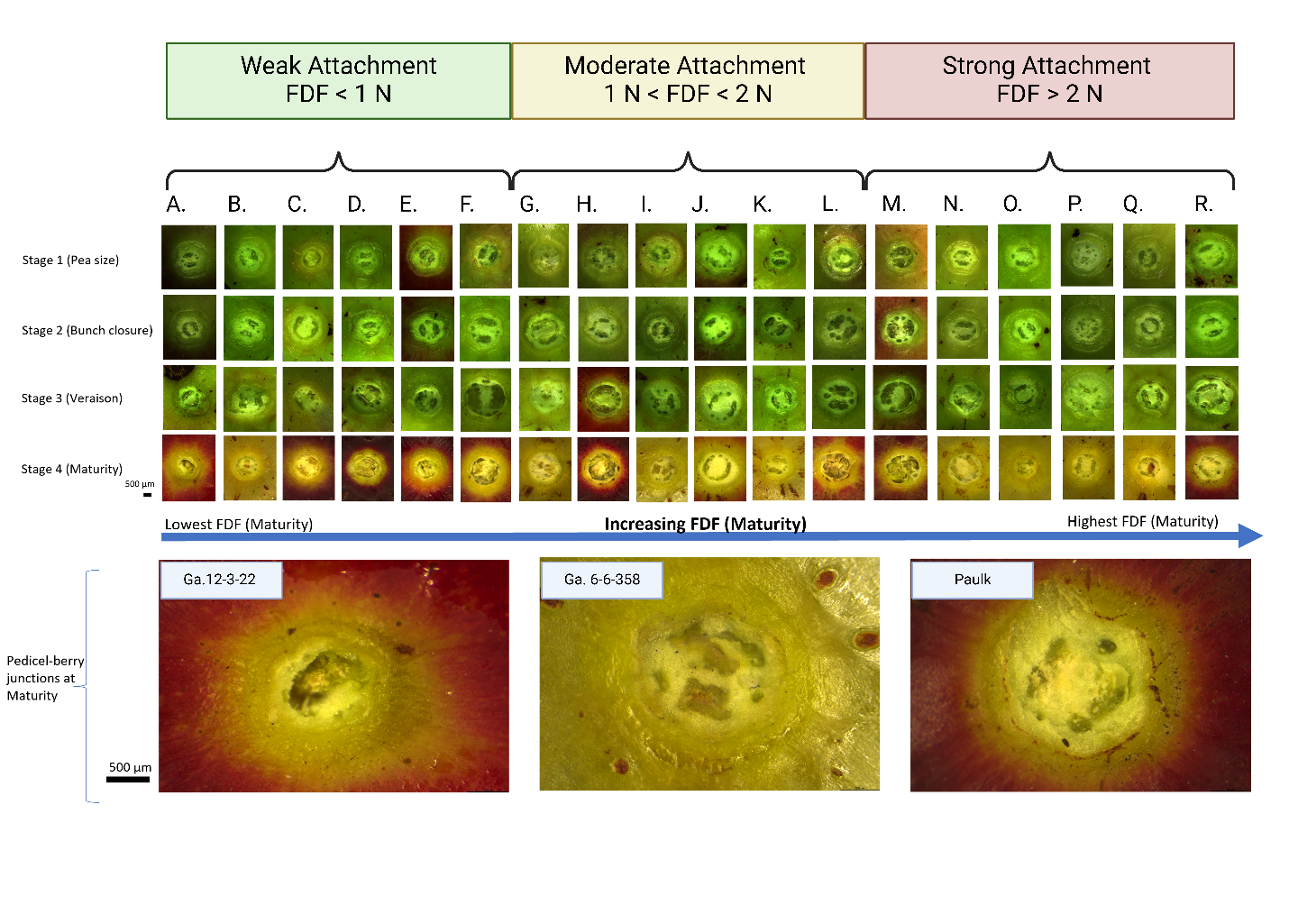


**Figure S3.** Light microscope images of tissue breakdown at the fruit-pedicel junction for all eighteen genotypes across four developmental stages. Images are ordered by increasing fruit detachment force from left to right and developmental stage from top to bottom. The genotypes are in the following order: (A) Ga. 12-3-33, (B) ‘Triumph’, (C) ‘Noble’, (D) ‘Alachua’, (E) Ga. 12-5-46, (F) Ga. 10-1-294, (G) ‘Carlos’, (H) Ga. 10-1-222, (I) Ga. 6-6-358, (J) ‘Supreme’, (K) Ga. 13-4-2, (L) Ga. 10-1-329, (M) Ga. 13-4-79, (O) ‘Granny Val’, (P) Ga. 8-1-12, (Q) Ga. 6-1-269, (R) ‘Hall’, (S) ‘Paulk’. The scale bars are 500 µm.


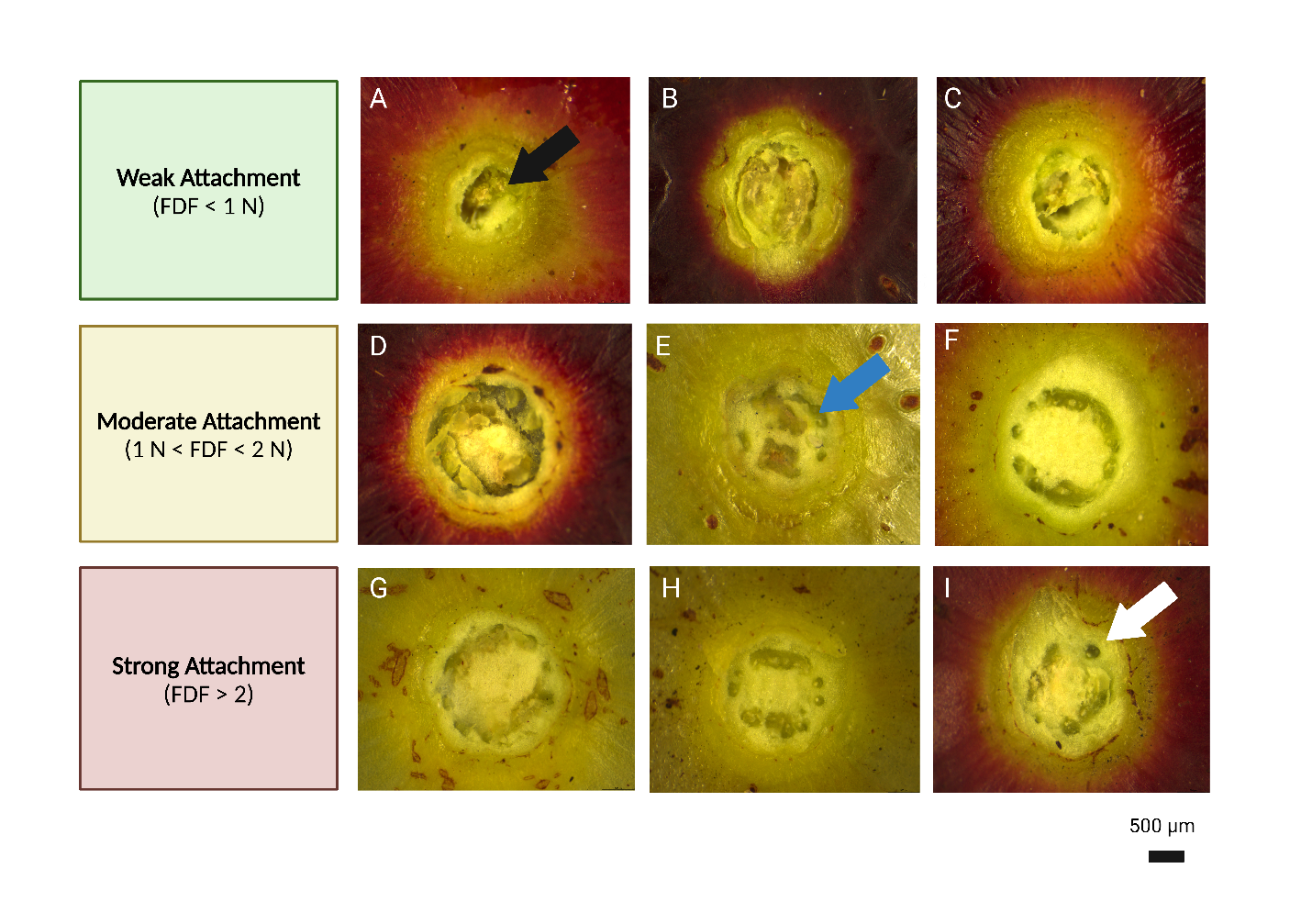


**Figure S4**. Fruit-pedicel junction across select genotypes throughout fruit development. Genotypes are ordered by increasing FDF at maturity. The genotypes displaying weak attachment (FDF < 1 N) are on the left: (A) Ga. 12-3-22, (B) ‘Alachua’, (C) Ga. 12-5-46. Genotypes displaying moderate attachment (1 N < FDF < 2 N) are in the middle: (D) Ga. 10-1-222, (E) Ga. 6-6-358, (F) ‘Supreme’. Genotypes displaying strong attachment (FDF > 2 N): (G) ‘Granny Val’, (H) Ga. 6-1-269, (I) ‘Paulk’. Rows from top to bottom are ordered by increasing developmental stage. Scale bars are 500 µm. The white arrow indicates an intact vascular bundle, the blue arrow indicates partial vascular bundle maintenance, and the black arrow indicates total vascular bundle breakdown (no vascular bundle maintenance). Figure created with BioRender.com.


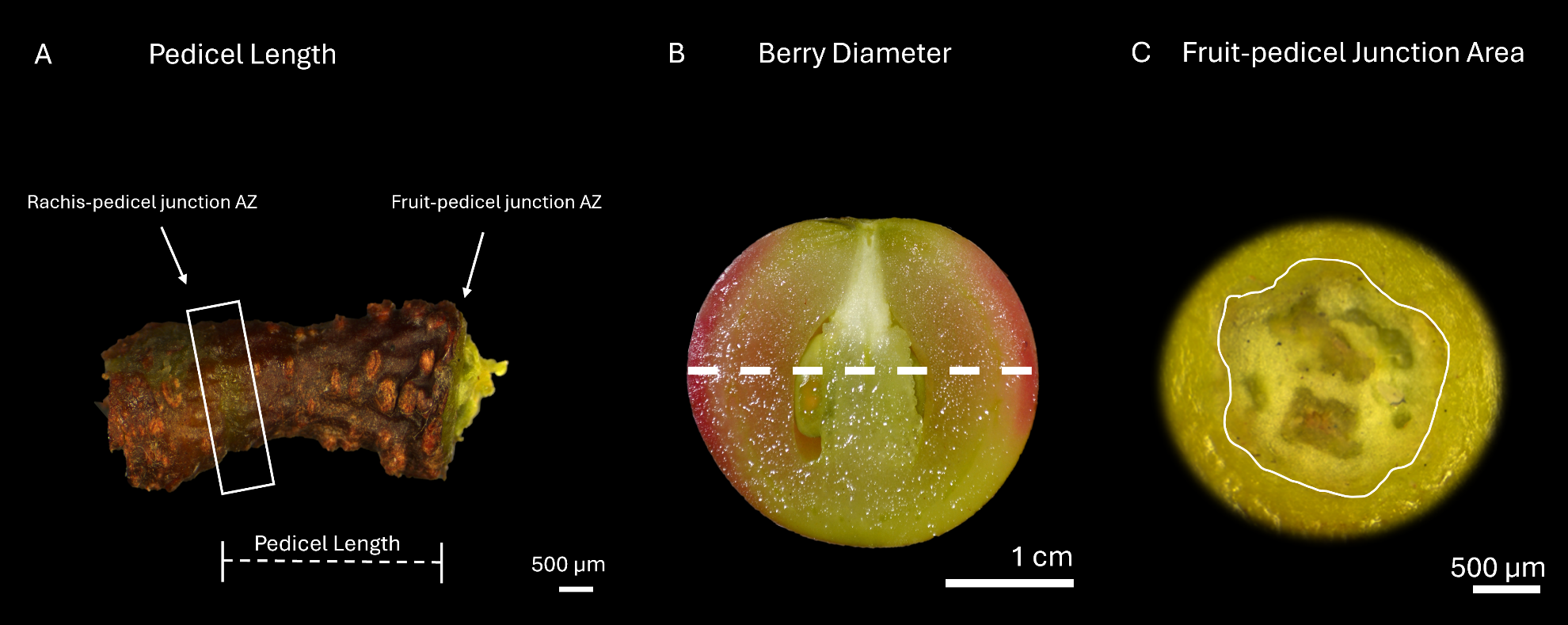


**Figure S5.** Diagram with guidelines for measuring (A) pedicel length, (B) Berry Diameter, and (C) Fruit-pedicel Junction Area.


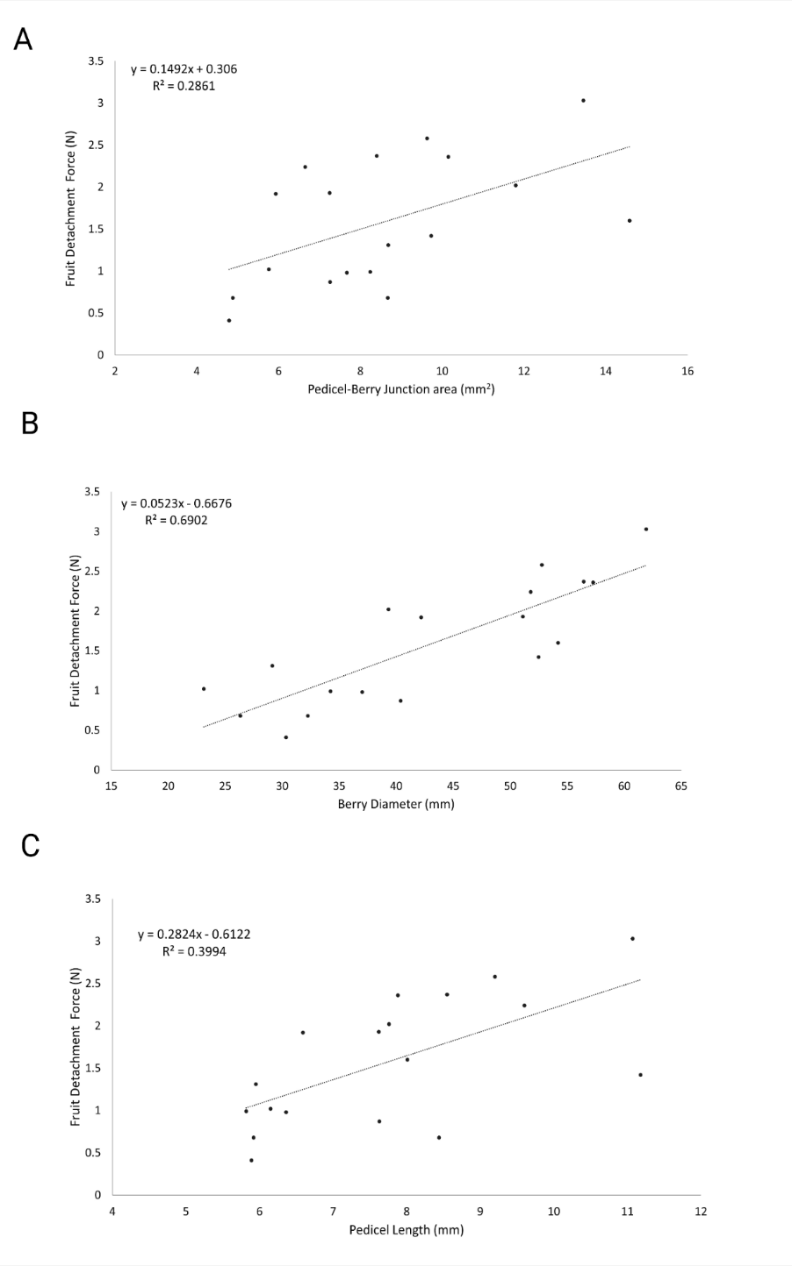


**Figure S6.** Linear regression plots comparing fruit detachment force (N) to (A) Fruit-pedicel junction area (mm^2^), p = 0.0222 (B) berry diameter (mm), p = 1.96E-05 (C) pedicel length (mm), p = 0.0049. All measurements were taken using mature, stage 4 berries (n=5).





**Figure S7.** RT-qPCR results as shown as the log2FC of expression from the pea-sized stage to maturity in both Ga.12-3-22 (W) and Ga. 6-1-269 (S). Expression is normalized to actin as an internal control. The Genes were selected based on their expression pattens and gene definitions. Log2FC detected through RNA-seq is shown in blue. Log2FC detected through RT-qPCR is shown in orange.


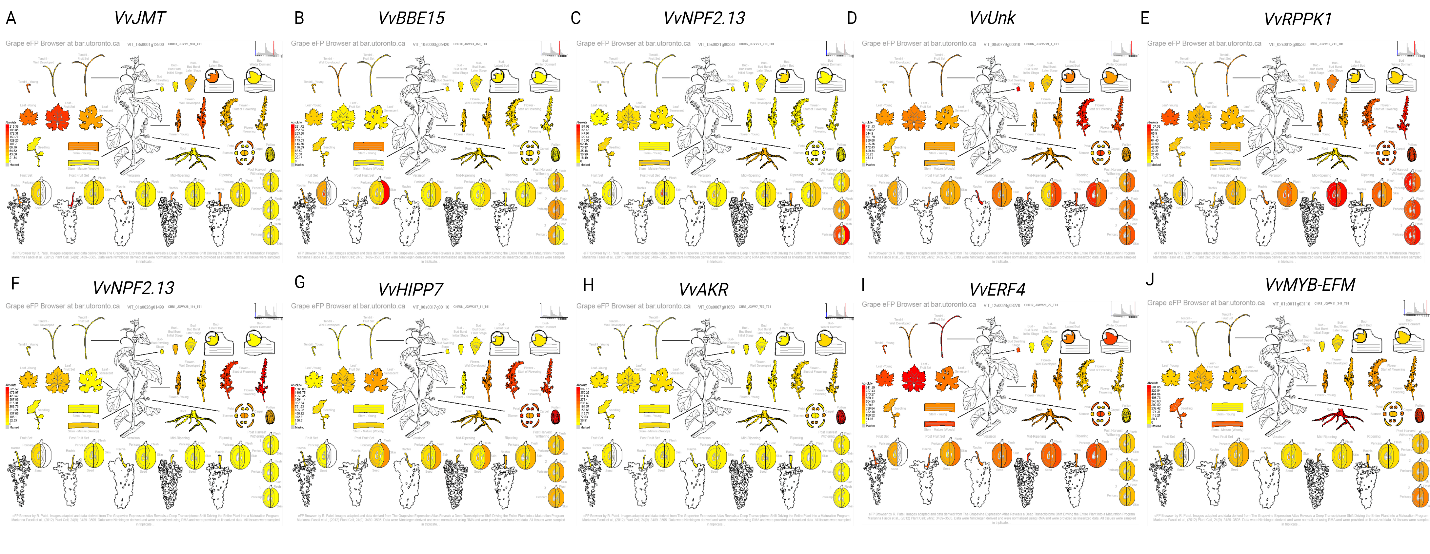


**Figure S8.** Electronic fluorescent pictographs (eFP) images showing spatial and temporal expression of genes that are oppositely regulated across the strongly and weakly attached genotypes. The eFPs are shown by gene in the following order: (A) VIT_18S0001G12900  (VvJMT) (B) VIT_10S0003G05420  (VvBBE15) , (C) VIT_15S0021G00330  (VvNPF7.3), (D) VIT_00s0779g00010 (VvUnk)), (E) VIT_02S0012G00530  (VvRPPK1), (F) VIT_01S0026G01490  (VvNPF2.13), (G) VIT_00S0317G00110  (VvHIPP7), (H) VIT_08S0007G01050  (VvAKR), (I) VIT_12S0028G03270  (VvERF4), (J) VIT_01S0011G03110  (VvMYB-EFM).


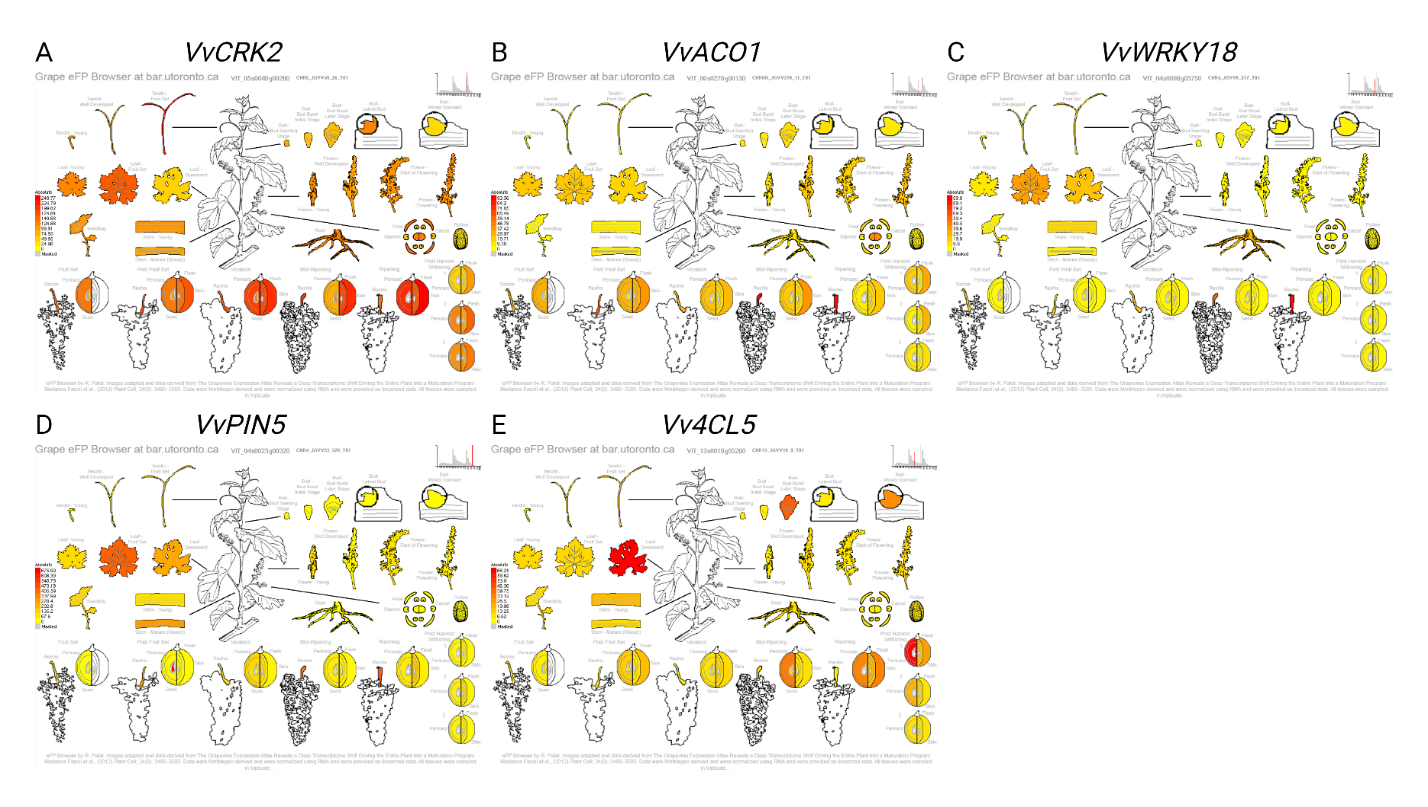


**Figure S9.** Electronic fluorescent pictographs (eFP) images showing spatial and temporal expression of genes that are up-regulated in Ga. 6-1-269 (S). The eFPs are shown by gene in the following order: (A) *VIT_05s0049g00280* (*VvCRK2*) (B) *VIT_00s0270g00130* (*VvACO1*), (C) *VIT_04S0008G05750* (*VvWRKY18*), (D) *VIT_04S0023G00320* (*VvPIN5*), (E) *VIT_13S0019G05260* (*Vv4CL5*).


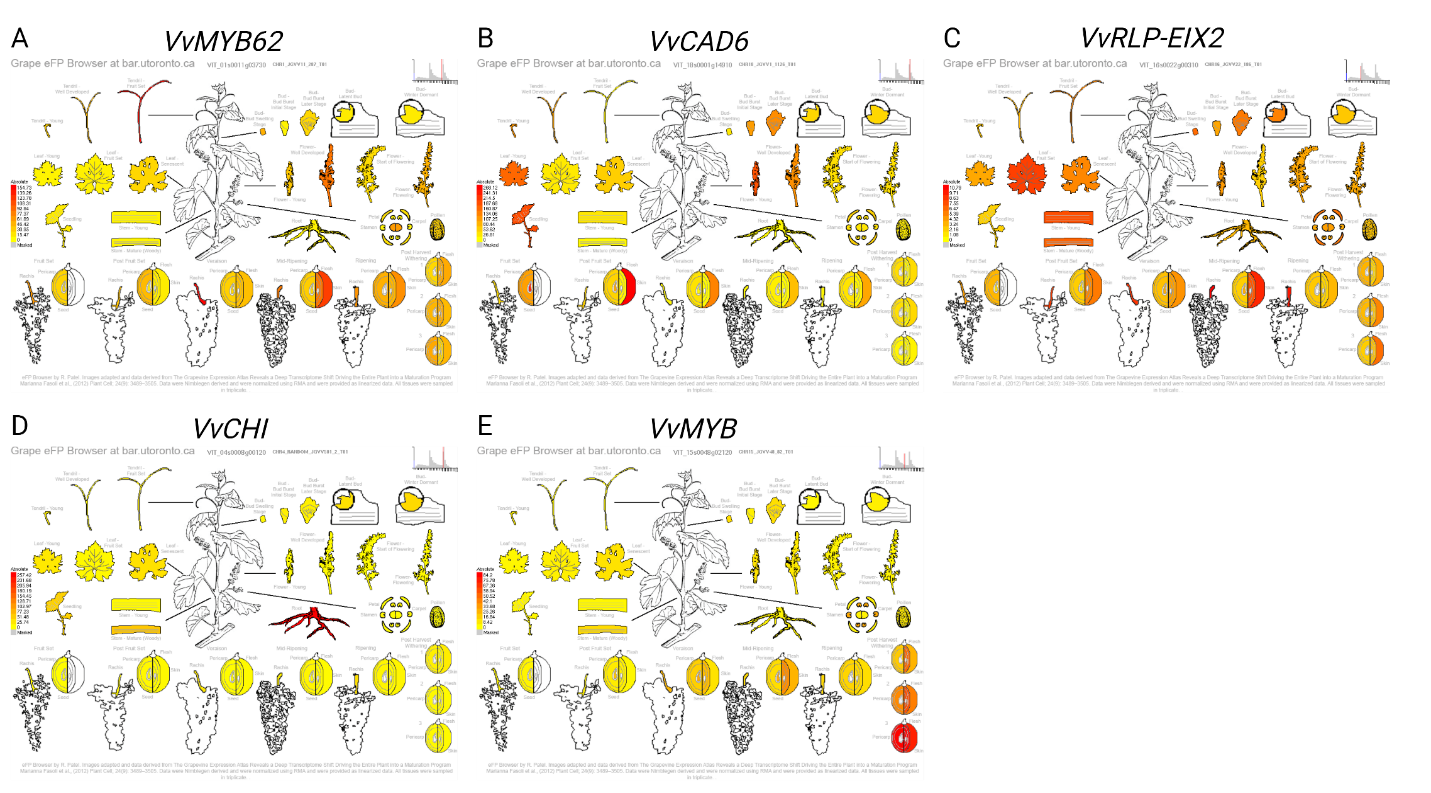


**Figure S10.** Electronic fluorescent pictographs (eFP) images showing spatial and temporal expression of genes that are up-regulated in Ga. 12-3-22 (W). The eFPs are shown by gene in the following order: (A) *VIT_01s0011g03730* (*VvMYB62*), (B) *VIT_18S0001G14910* (*VvCAD6*), (C) *VIT_16s0022g00310* (*VvRLP*-*EIX2*), (D) *VIT_04s0008g00120* (*VvCHI*), (E) *VIT_15s0048g02120* (*VvMYB*).


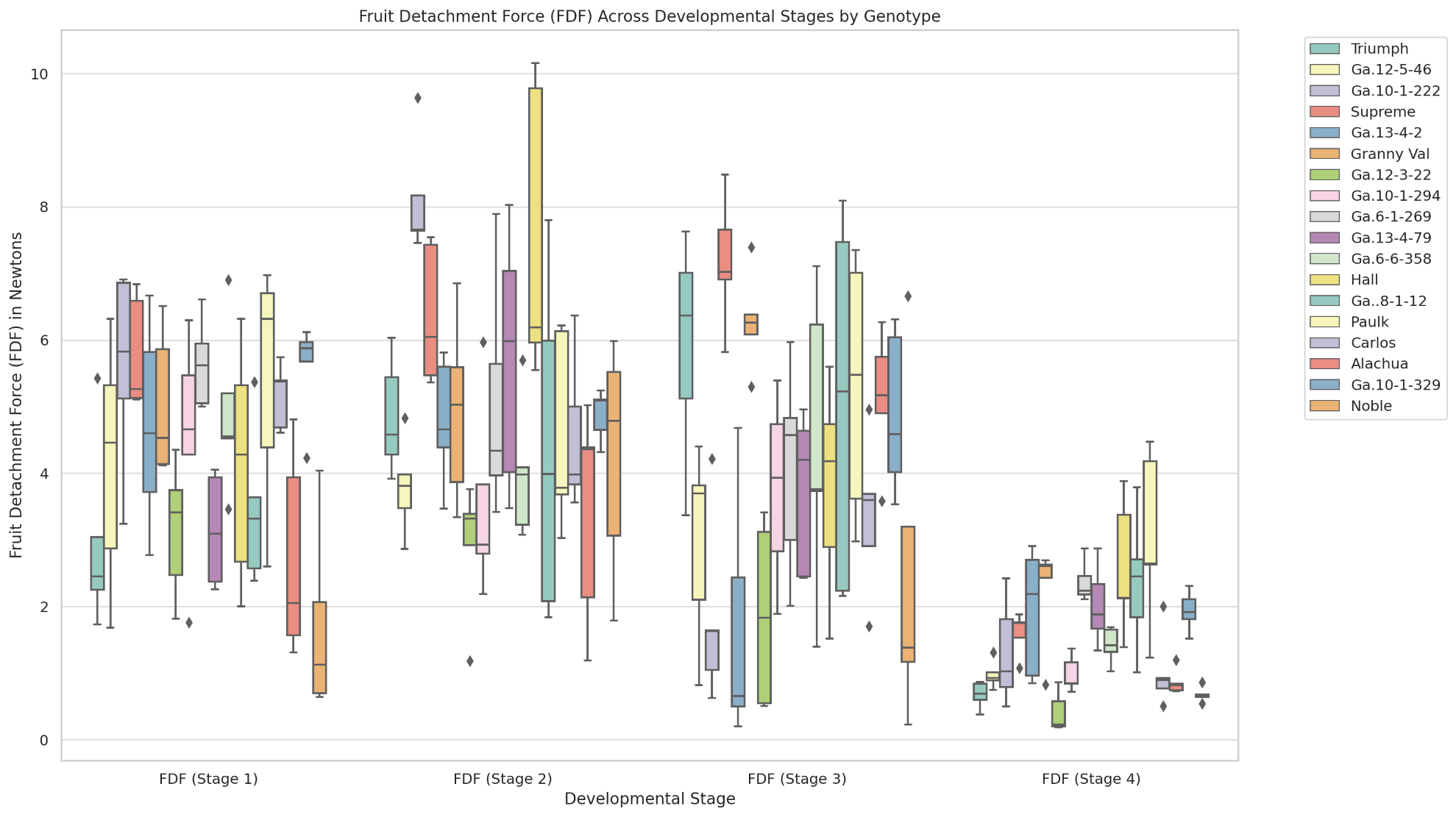


**Figure S11.** Boxplots showing the spread of Fruit Detachment Force (FDF) data for each genotype across the four developmental stages. Different color boxplots indicate different genotypes.Graph shows highest variability at Stage 3 and lowest variability at Stage 4. X-axis shows developmental stage, Y-axis shows FDF in Newtons. N=5 for each genotype and developmental stage.

**Table S1.** University of Georgia-developed varieties used in this study and their characteristics.

| **UGA Label** | **Characteristics** |
| --- | --- |
| Ga. 8-1-12 | Self-fertile bronze. |
| Ga. 6-1-269 | Self-fertile bronze. |
| Ga. 6-6-358 | Self-fertile bronze. |
| Ga. 12-3-22 | Self-fertile red. |
| Ga. 10-1-329 | Self-fertile black. |
| Ga. 12-5-46 | Self-fertile black. |
| Ga. 10-1-294 | Self-fertile black. |
| Ga. 10-1-222 | Self-fertile black. |
| Ga. 13-4-79 | Self-fertile black. |
| Ga. 13-4-2 | Self-fertile bronze. |

**Table S2.** Mean Pedicel-Fruit Junction (mm^2^), berry diameter (mm), pedicel length (mm), and fruit detachment force (N) for all eighteen genotypes at maturity. Error is shown as standard error.

| **Genotype** | **Pedicel-Fruit Junction area** | **Berry Diameter** | **Pedicel Length** | **Fruit Detachment Force** |
| --- | --- | --- | --- | --- |
|  | **(mm^2^)** | **(mm)** | **(mm)** | **(N)** |
|  |  |  |  |  |
| Ga. 12-3-22 | 4.79 ± 0.91 | 30.33 ± 1.23 | 5.89 ± 1.08 | 0.41 ± 0.30 |
| Triumph | 4.88 ± 0.74 | 26.32 ± 1.00 | 5.92 ± 1.24 | 0.68 ± 0.20 |
| Noble | 8.67 ± 0.83 | 32.24 ± 0.16 | 8.44 ± 1.00 | 0.68 ± 0.12 |
| Alachua | 7.26 ± 0.67 | 40.39 ± 2.95 | 7.63 ± 1.49 | 0.87 ± 0.19 |
| Ga. 12-5-46 | 7.67 ± 1.39 | 37.02 ± 0.74 | 6.36 ± 0.86 | 0.98 ± 0.21 |
| Ga. 10-1-294 | 8.24 ± 0.30 | 34.23 ± 1.37 | 5.82 ± 0.50 | 0.99 ± 0.27 |
| Carlos | 5.76 ± 1.96 | 23.12 ± 0.58 | 6.15 ± 0.76 | 1.02 ± 0.57 |
| Ga. 10-1-222 | 8.68 ± 1.88 | 29.13 ± 2.26 | 5.95 ± 0.78 | 1.31 ± 0.79 |
| Ga. 6-6-358 | 9.73 ± 2.23 | 52.49 ± 1.87 | 11.18 ± 1.19 | 1.42 ± 0.27 |
| Supreme | 14.58 ± 1.87 | 54.20 ± 3.07 | 8.01 ± 1.15 | 1.60 ± 0.32 |
| Ga. 13-4-2 | 5.93 ± 1.53 | 42.18 ± 2.10 | 6.59 ± 1.15 | 1.92 ± 0.97 |
| Ga. 10-1-329 | 7.25 ± 1.79 | 51.10 ± 2.26 | 7.62 ± 0.99 | 1.93 ± 0.30 |
| Ga. 13-4-79 | 11.80 ± 2 .28 | 39.31 ± 3.41 | 7.76 ± 1.01 | 2.02 ± 0.60 |
| Granny Val | 6.65 ± 1.04 | 51.79 ± 2.09 | 9.60 ± 1.05 | 2.24 ± 0.79 |
| Ga. 8-1-12 | 10.15 ± 1.65 | 57.27 ± 0.96 | 7.88 ± 1.19 | 2.36 ± 1.03 |
| Ga. 6-1-269 | 8.40 ± 1.57 | 56.44 ± 3.15 | 8.55 ± 0.86 | 2.37 ± 0.31 |
| Hall | 9.63 ± 0.92 | 52.77 ± 4.71 | 9.20 ± 0.96 | 2.58 ± 1.02 |
| Paulk | 13.45 ± 2.12 | 61.93 ± 2.30 | 11.07 ± 1.19 | 3.03 ± 1.32 |

**Table S3.** Genes that are both up-regulated or both down-regulated in Ga.12-3-22 and Ga.6-1-269 with a Log2FC ≥ 2 and a p-value ≤ 0.05.

|  |  |  | **Ga.6-1-269** | **Ga.12-3-22** |  |
| --- | --- | --- | --- | --- | --- |
| **Ensembl Gene ID** | **Putative ATH Orthologue** | **NCBI Protein ID** | **Fold change (log2)** | **Fold change (log2)** | **Gene Annotation** |
| *Up-regulated in both Ga.12-3-.22 (W) and Ga.6-1-269 (S)* | | | | | |
| *VIT_04S0044G00380* | *AT1G25270.1* | XP_002271832.2 | 7.18 | 10.02 | WAT1-related protein At1g68170-like |
| *VIT_10S0003G02140* | *AT5G45660.1* | XP_019078008.1 | 2.03 | 7.44 | - |
| *VIT_03S0063G01690* | *AT4G31940.1* | XP_002284806.1 | 4.73 | 4.75 | cytochrome P450 CYP82D47 |
| *VIT_02S0033G00300* | *AT5G45580.2* | XP_002264257.2 | 2.7 | 4.37 | myb family transcription factor PHL11 |
| *VIT_00S0567G00060* | *AT2G26560.1* | XP_002262942.1 | 3.52 | 3.99 | patatin-like protein 2 |
| *VIT_02S0025G03120* | *AT1G75500.1* | XP_002271326.3 | 5.1 | 3.62 | WAT1-related protein At1g43650 |
| *VIT_03S0091G00500* | *AT3G19550.1* | XP_019074178.1 | 4.12 | 3.49 | - |
| *VIT_01S0010G02330* | *AT3G26040.1* | XP_002267080.1 | 4.55 | 3.26 | vinorine synthase |
| *VIT_02S0025G01630* | *AT5G24270.4* | NP_001268186.1 | 3.08 | 3.25 | calcineurin B-like protein 7 |
| *VIT_02S0025G00700* | *AT3G18440.1* | XP_002272229.1 | 3.92 | 3.13 | aluminum-activated malate transporter 9-like |
| *VIT_04S0008G07080* | *AT5G10770.1* | XP_002283470.1 | 2.93 | 2.94 | aspartyl protease family protein At5g10770-like |
| *VIT_02S0025G03140* | *AT4G08290.1* | XP_002273800.3 | 4.01 | 2.84 | WAT1-related protein At1g43650-like |
| *VIT_16S0050G02540* | *AT5G53190.1* | XP_002267886.1 | 3.1 | 2.72 | bidirectional sugar transporter SWEET3 |
| *VIT_19S0085G00740* | *AT3G12750.1* | XP_002264603.2 | 4.23 | 2.7 | zinc transporter 1-like |
| *VIT_00S0480G00060* | *-* | NP_001268045.1 | 6.23 | 2.48 | "polyphenol oxidase |
| *VIT_03S0091G00490* | *AT3G19550.1* | XP_002262812.1 | 3.2 | 2.45 | - |
| *VIT_18S0001G08200* | *AT1G58340.1* | XP_002283609.2 | 4.27 | 2.39 | protein DETOXIFICATION 48 |
| *VIT_07S0191G00080* | *AT5G65280.1* | XP_002262692.1 | 2.87 | 2.35 | lanC-like protein GCL1 |
| *VIT_09S0002G02610* | *AT1G73040.1* | XP_010654564.1 | 3.44 | 2.32 | jacalin-related lectin 19 |
| *VIT_16S0050G01860* | *AT5G24030.1* | XP_010662648.1 | 3.84 | 2.3 | S-type anion channel SLAH2 isoform X2 |
| *VIT_01S0011G02350* | *AT1G23980.1* | XP_010648283.1 | 8.45 | 2.28 | E3 ubiquitin-protein ligase RNF181-like |
| *VIT_02S0025G03010* | *AT2G18196.1* | XP_002277877.2 | 3.96 | 2.27 | heavy metal-associated isoprenylated plant protein 30 |
| *VIT_11S0052G00280* | *AT2G34690.1* | XP_010656881.1 | 3.68 | 2.18 | accelerated cell death 11 |
| *VIT_03S0017G01740* | *AT2G38760.1* | XP_002268873.2 | 5.72 | 2.15 | annexin D3 |
| *VIT_18S0001G03170* | *AT4G08290.1* | XP_002284458.1 | 4.77 | 2.14 | WAT1-related protein At4g08290 isoform X1 |
| *VIT_08S0007G07580* | *AT3G04030.3* | XP_010653833.1 | 4.08 | 2.07 | myb-related protein 2-like |
| *Down-regulated in both Ga.12-3-22 (W) and Ga.6-1-269 (S)* | | | | | |
| *VIT_12S0142G00040* | *AT1G06520.1* | XP_010656931.2 | -7.32 | -5.75 | glycerol-3-phosphate acyltransferase 1 isoform X2 |
| *VIT_13S0019G03360* | *AT2G38080.1* | XP_002265173.1 | -5.42 | -6.75 | laccase-4-like |
| *VIT_05S0102G00880* | *AT3G59530.3* | XP_003632076.1 | -4.82 | -7.52 | protein STRICTOSIDINE SYNTHASE-LIKE 13 |
| *VIT_00S0527G00030* | *AT2G34700.1* | XP_002274293.4 | -4.53 | -3.41 | non-classical arabinogalactan protein 31 |
| *VIT_07S0151G00760* | *AT3G22600.1* | XP_002282896.2 | -4.19 | -3.21 | non-specific lipid-transfer protein-like protein At2g13820 isoform X1 |
| *VIT_07S0031G00110* | *AT5G67210.1* | XP_002283187.1 | -3.92 | -3.82 | protein IRX15-LIKE |
| *VIT_17S0000G05810* | *AT5G15130.1* | XP_010663394.1 | -3.88 | -2.08 | probable WRKY transcription factor 72 isoform X1 |
| *VIT_03S0088G00110* | *AT1G33540.1* | XP_002266615.3 | -3.54 | -6.49 | serine carboxypeptidase-like 17 |
| *VIT_14S0083G01100* | *AT3G08900.1* | XP_002281356.1 | -3.47 | -2.68 | UDP-arabinopyranose mutase 3 |
| *VIT_03S0063G02320* | *AT1G49320.1* | XP_003631606.1 | -3.27 | -2.02 | BURP domain-containing protein BNM2C-like |
| *VIT_16S0050G01880* | *AT5G24080.2* | XP_002275726.1 | -3.2 | -6.58 | G-type lectin S-receptor-like serine/threonine-protein kinase At5g24080 isoform X1 |
| *VIT_00S0302G00010* | *AT4G35730.1* | XP_003635529.1 | -3.04 | -6.87 | IST1-like protein |
| *VIT_14S0081G00060* | *AT2G30570.1* | XP_002264508.1 | -3.03 | -2.92 | "photosystem II reaction center W protein |
| *VIT_12S0028G02240* | *AT2G42580.1* | XP_002276519.2 | -3.03 | -7.45 | inactive TPR repeat-containing thioredoxin TTL3 isoform X1 |
| *VIT_07S0255G00030* | *AT2G23540.1* | XP_003632456.1 | -2.98 | -2.78 | GDSL esterase/lipase At2g23540 |
| *VIT_05S0020G02260* | *AT5G18840.1* | XP_002278654.3 | -2.92 | -7.21 | sugar transporter ERD6-like 7 isoform X2 |
| *VIT_15S0021G01390* | *AT3G60270.1* | XP_002272263.1 | -2.89 | -2.76 | uclacyanin-3 |
| *VIT_18S0001G12580* | *AT2G02540.2* | XP_002281371.1 | -2.88 | -7.01 | zinc-finger homeodomain protein 6 |
| *VIT_12S0059G01000* | *AT2G32530.1* | XP_003633282.1 | -2.7 | -2.35 | cellulose synthase-like protein H1 |
| *VIT_07S0005G01030* | *AT1G02730.1* | XP_002274010.1 | -2.69 | -6.94 | cellulose synthase-like protein D5 |
| *VIT_13S0064G00480* | *AT2G44480.3* | XP_002273684.2 | -2.64 | -3.04 | furcatin hydrolase-like |
| *VIT_12S0057G00430* | *AT5G47530.1* | XP_002273239.1 | -2.33 | -4.32 | cytochrome b561 and DOMON domain-containing protein At5g47530 |
| *VIT_18S0001G14450* | *AT1G20020.1* | XP_002274366.2 | -2.25 | -8.84 | "ferredoxin--NADP reductase |
| *VIT_01S0026G01340* | *AT1G10370.1* | XP_019076242.1 | -2.18 | -2.82 | uncharacterized protein LOC117920146 |
| *VIT_18S0001G05530* | *AT1G35720.1* | XP_002285795.1 | -2.18 | -2.58 | annexin D1 |
| *VIT_04S0008G00530* | *AT4G32830.1* | XP_002275382.1 | -2.1 | -4.8 | serine/threonine-protein kinase Aurora-1 |

**Table S4.** All transcription factors identified in Ga. 6-1-269 (S) and Ga.12-3-22 (W). Table is separated into up and down-regulated per genotype. Shown are the transcription factor family, the gene id, and the transcript id. Gene id and transcript id are from Ensembl plants database. Shown are transcription factors with a Log2FC ≥ 1 and a p-value ≤ 0.05 are shown.

| Transcription Factor Family | Gene ID\|Transcript ID (Ensembl) |
| --- | --- |
| *Up-regulated in Ga.6-1-269 (S)* | |
| ARF | *Vitvi06g00272\|Vitvi06g00272_t001* |
| ARF | *Vitvi06g00272\|Vitvi06g00272_t002* |
| B3 | *Vitvi07g00781\|Vitvi07g00781_t001* |
| B3 | *Vitvi17g01015\|Vitvi17g01015_t001* |
| bHLH | *Vitvi01g00876\|Vitvi01g00876_t001* |
| bHLH | *Vitvi01g01316\|Vitvi01g01316_t001* |
| bHLH | *Vitvi01g01316\|Vitvi01g01316_t002* |
| bHLH | *Vitvi01g01316\|Vitvi01g01316_t004* |
| bHLH | *Vitvi01g01482\|Vitvi01g01482_t001* |
| bHLH | *Vitvi04g01312\|Vitvi04g01312_t001* |
| bHLH | *Vitvi04g01361\|Vitvi04g01361_t001* |
| bHLH | *Vitvi04g01662\|Vitvi04g01662_t001* |
| bHLH | *Vitvi05g01597\|Vitvi05g01597_t001* |
| bHLH | *Vitvi07g00046\|Vitvi07g00046_t001* |
| bHLH | *Vitvi08g00719\|Vitvi08g00719_t001* |
| bHLH | *Vitvi08g01856\|Vitvi08g01856_t001* |
| bHLH | *Vitvi14g00477\|Vitvi14g00477_t001* |
| bHLH | *Vitvi14g00562\|Vitvi14g00562_t001* |
| bHLH | *Vitvi15g01124\|Vitvi15g01124_t001* |
| bHLH | *Vitvi17g00046\|Vitvi17g00046_t001* |
| bHLH | *Vitvi17g00795\|Vitvi17g00795_t001* |
| bHLH | *Vitvi18g00617\|Vitvi18g00617_t001* |
| bHLH | *Vitvi18g02008\|Vitvi18g02008_t001* |
| bHLH | *Vitvi19g00367\|Vitvi19g00367_t001* |
| bZIP | *Vitvi03g00292\|Vitvi03g00292_t001* |
| bZIP | *Vitvi08g00950\|Vitvi08g00950_t001* |
| bZIP | *Vitvi13g01609\|Vitvi13g01609_t001* |
| bZIP | *Vitvi14g00094\|Vitvi14g00094_t001* |
| bZIP | *Vitvi14g01302\|Vitvi14g01302_t001* |
| bZIP | *Vitvi15g01027\|Vitvi15g01027_t001* |
| bZIP | *Vitvi15g01027\|Vitvi15g01027_t002* |
| bZIP | *Vitvi18g00930\|Vitvi18g00930_t001* |
| bZIP | *Vitvi18g00930\|Vitvi18g00930_t002* |
| bZIP | *Vitvi18g00930\|Vitvi18g00930_t003* |
| bZIP | *Vitvi18g02978\|Vitvi18g02978_t001* |
| C2H2 | *Vitvi01g00529\|Vitvi01g00529_t001* |
| C2H2 | *Vitvi11g01259\|Vitvi11g01259_t001* |
| C2H2 | *Vitvi17g00157\|Vitvi17g00157_t001* |
| C2H2 | *Vitvi18g00755\|Vitvi18g00755_t001* |
| C3H | *Vitvi04g01157\|Vitvi04g01157_t001* |
| C3H | *Vitvi04g01157\|Vitvi04g01157_t002* |
| C3H | *Vitvi08g01732\|Vitvi08g01732_t001* |
| C3H | *Vitvi12g00449\|Vitvi12g00449_t001* |
| C3H | *Vitvi14g02452\|Vitvi14g02452_t001* |
| CO-like | *Vitvi14g01296\|Vitvi14g01296_t001* |
| CO-like | *Vitvi17g00328\|Vitvi17g00328_t001* |
| DBB | *Vitvi04g01423\|Vitvi04g01423_t001* |
| Dof | *Vitvi01g01026\|Vitvi01g01026_t001* |
| Dof | *Vitvi08g01186\|Vitvi08g01186_t001* |
| Dof | *Vitvi10g00479\|Vitvi10g00479_t001* |
| Dof | *Vitvi15g00936\|Vitvi15g00936_t001* |
| EIL | *Vitvi06g01036\|Vitvi06g01036_t001* |
| ERF | *Vitvi03g00500\|Vitvi03g00500_t001* |
| ERF | *Vitvi04g00190\|Vitvi04g00190_t001* |
| ERF | *Vitvi05g00334\|Vitvi05g00334_t001* |
| ERF | *Vitvi05g01073\|Vitvi05g01073_t001* |
| ERF | *Vitvi06g00052\|Vitvi06g00052_t001* |
| ERF | *Vitvi06g00810\|Vitvi06g00810_t001* |
| ERF | *Vitvi07g00357\|Vitvi07g00357_t001* |
| ERF | *Vitvi07g02062\|Vitvi07g02062_t001* |
| ERF | *Vitvi07g02067\|Vitvi07g02067_t001* |
| ERF | *Vitvi08g01966\|Vitvi08g01966_t001* |
| ERF | *Vitvi10g00521\|Vitvi10g00521_t001* |
| ERF | *Vitvi10g00522\|Vitvi10g00522_t001* |
| ERF | *Vitvi11g00045\|Vitvi11g00045_t001* |
| ERF | *Vitvi12g00274\|Vitvi12g00274_t001* |
| ERF | *Vitvi13g00080\|Vitvi13g00080_t001* |
| ERF | *Vitvi14g00564\|Vitvi14g00564_t001* |
| ERF | *Vitvi14g01441\|Vitvi14g01441_t001* |
| ERF | *Vitvi15g01021\|Vitvi15g01021_t001* |
| ERF | *Vitvi16g00350\|Vitvi16g00350_t001* |
| ERF | *Vitvi16g00362\|Vitvi16g00362_t001* |
| ERF | *Vitvi16g00370\|Vitvi16g00370_t001* |
| ERF | *Vitvi16g00380\|Vitvi16g00380_t001* |
| ERF | *Vitvi16g01429\|Vitvi16g01429_t001* |
| ERF | *Vitvi16g04340\|Vitvi16g04340_t001* |
| ERF | *Vitvi18g02191\|Vitvi18g02191_t001* |
| ERF | *Vitvi18g02240\|Vitvi18g02240_t001* |
| ERF | *Vitvi18g02241\|Vitvi18g02241_t001* |
| ERF | *Vitvi18g02398\|Vitvi18g02398_t001* |
| ERF | *Vitvi19g00260\|Vitvi19g00260_t001* |
| ERF | *Vitvi00g04528\|Vitvi00g04528_t001* |
| FAR1 | *Vitvi12g00713\|Vitvi12g00713_t001* |
| G2-like | *Vitvi01g00249\|Vitvi01g00249_t001* |
| G2-like | *Vitvi02g01004\|Vitvi02g01004_t001* |
| G2-like | *Vitvi07g01325\|Vitvi07g01325_t001* |
| G2-like | *Vitvi08g00955\|Vitvi08g00955_t001* |
| G2-like | *Vitvi08g01723\|Vitvi08g01723_t001* |
| G2-like | *Vitvi08g01834\|Vitvi08g01834_t001* |
| G2-like | *Vitvi08g01834\|Vitvi08g01834_t002* |
| G2-like | *Vitvi19g01742\|Vitvi19g01742_t001* |
| GATA | *Vitvi04g00289\|Vitvi04g00289_t001* |
| GATA | *Vitvi05g00077\|Vitvi05g00077_t001* |
| GeBP | *Vitvi12g00561\|Vitvi12g00561_t001* |
| GeBP | *Vitvi19g01979\|Vitvi19g01979_t001* |
| GRAS | *Vitvi04g01247\|Vitvi04g01247_t001* |
| GRAS | *Vitvi05g01554\|Vitvi05g01554_t001* |
| GRAS | *Vitvi06g00492\|Vitvi06g00492_t001* |
| GRAS | *Vitvi07g00627\|Vitvi07g00627_t001* |
| GRAS | *Vitvi12g00571\|Vitvi12g00571_t001* |
| GRAS | *Vitvi13g01556\|Vitvi13g01556_t001* |
| GRAS | *Vitvi13g01556\|Vitvi13g01556_t002* |
| GRAS | *Vitvi19g00932\|Vitvi19g00932_t001* |
| HD-ZIP | *Vitvi01g00958\|Vitvi01g00958_t001* |
| HD-ZIP | *Vitvi01g00958\|Vitvi01g00958_t002* |
| HD-ZIP | *Vitvi02g00228\|Vitvi02g00228_t001* |
| HD-ZIP | *Vitvi02g01717\|Vitvi02g01717_t001* |
| HD-ZIP | *Vitvi07g01488\|Vitvi07g01488_t001* |
| HD-ZIP | *Vitvi15g00579\|Vitvi15g00579_t001* |
| HSF | *Vitvi02g00387\|Vitvi02g00387_t001* |
| HSF | *Vitvi02g00387\|Vitvi02g00387_t002* |
| HSF | *Vitvi02g00387\|Vitvi02g00387_t003* |
| HSF | *Vitvi06g01878\|Vitvi06g01878_t001* |
| HSF | *Vitvi07g01749\|Vitvi07g01749_t001* |
| HSF | *Vitvi16g00982\|Vitvi16g00982_t001* |
| LBD | *Vitvi01g00291\|Vitvi01g00291_t001* |
| LBD | *Vitvi07g00572\|Vitvi07g00572_t001* |
| LBD | *Vitvi07g01610\|Vitvi07g01610_t001* |
| LBD | *Vitvi09g00188\|Vitvi09g00188_t001* |
| LBD | *Vitvi10g01237\|Vitvi10g01237_t001* |
| LBD | *Vitvi13g00109\|Vitvi13g00109_t001* |
| LBD | *Vitvi13g00549\|Vitvi13g00549_t001* |
| LBD | *Vitvi13g00552\|Vitvi13g00552_t001* |
| LBD | *Vitvi17g00890\|Vitvi17g00890_t001* |
| LBD | *Vitvi17g00890\|Vitvi17g00890_t002* |
| LBD | *Vitvi19g01589\|Vitvi19g01589_t001* |
| MIKC_MADS | *Vitvi07g01441\|Vitvi07g01441_t001* |
| MIKC_MADS | *Vitvi18g02133\|Vitvi18g02133_t001* |
| M-type_MADS | *Vitvi14g00026\|Vitvi14g00026_t001* |
| MYB | *Vitvi01g01028\|Vitvi01g01028_t001* |
| MYB | *Vitvi02g01019\|Vitvi02g01019_t001* |
| MYB | *Vitvi02g01308\|Vitvi02g01308_t001* |
| MYB | *Vitvi04g00153\|Vitvi04g00153_t001* |
| MYB | *Vitvi05g01733\|Vitvi05g01733_t001* |
| MYB | *Vitvi06g00592\|Vitvi06g00592_t001* |
| MYB | *Vitvi08g01336\|Vitvi08g01336_t001* |
| MYB | *Vitvi08g01336\|Vitvi08g01336_t002* |
| MYB | *Vitvi09g00110\|Vitvi09g00110_t001* |
| MYB | *Vitvi09g00142\|Vitvi09g00142_t001* |
| MYB | *Vitvi10g00345\|Vitvi10g00345_t001* |
| MYB | *Vitvi11g00097\|Vitvi11g00097_t001* |
| MYB | *Vitvi12g00625\|Vitvi12g00625_t001* |
| MYB | *Vitvi12g00626\|Vitvi12g00626_t001* |
| MYB | *Vitvi13g00510\|Vitvi13g00510_t001* |
| MYB | *Vitvi14g00925\|Vitvi14g00925_t001* |
| MYB | *Vitvi14g00974\|Vitvi14g00974_t001* |
| MYB | *Vitvi14g01615\|Vitvi14g01615_t001* |
| MYB | *Vitvi14g01960\|Vitvi14g01960_t001* |
| MYB | *Vitvi14g01960\|Vitvi14g01960_t002* |
| MYB | *Vitvi15g00594\|Vitvi15g00594_t001* |
| MYB | *Vitvi15g01161\|Vitvi15g01161_t001* |
| MYB | *Vitvi15g04655\|Vitvi15g04655_t001* |
| MYB | *Vitvi16g00098\|Vitvi16g00098_t001* |
| MYB | *Vitvi17g00231\|Vitvi17g00231_t001* |
| MYB_related | *Vitvi01g00867\|Vitvi01g00867_t001* |
| MYB_related | *Vitvi03g00318\|Vitvi03g00318_t001* |
| MYB_related | *Vitvi10g01608\|Vitvi10g01608_t001* |
| MYB_related | *Vitvi11g00197\|Vitvi11g00197_t001* |
| MYB_related | *Vitvi15g01097\|Vitvi15g01097_t001* |
| MYB_related | *Vitvi15g01097\|Vitvi15g01097_t002* |
| MYB_related | *Vitvi17g00726\|Vitvi17g00726_t001* |
| MYB_related | *Vitvi18g00973\|Vitvi18g00973_t001* |
| NAC | *Vitvi03g00207\|Vitvi03g00207_t001* |
| NAC | *Vitvi06g00305\|Vitvi06g00305_t001* |
| NAC | *Vitvi06g01515\|Vitvi06g01515_t001* |
| NAC | *Vitvi08g01052\|Vitvi08g01052_t001* |
| NAC | *Vitvi08g01426\|Vitvi08g01426_t001* |
| NAC | *Vitvi11g00241\|Vitvi11g00241_t002* |
| NAC | *Vitvi12g00076\|Vitvi12g00076_t001* |
| NAC | *Vitvi12g01655\|Vitvi12g01655_t001* |
| NAC | *Vitvi15g01540\|Vitvi15g01540_t001* |
| NAC | *Vitvi18g02011\|Vitvi18g02011_t001* |
| NAC | *Vitvi19g00271\|Vitvi19g00271_t001* |
| NAC | *Vitvi19g01564\|Vitvi19g01564_t001* |
| NAC | *Vitvi19g01751\|Vitvi19g01751_t001* |
| NAC | *Vitvi19g02272\|Vitvi19g02272_t001* |
| NF-YB | *Vitvi07g01823\|Vitvi07g01823_t001* |
| NF-YB | *Vitvi19g00673\|Vitvi19g00673_t001* |
| NF-YC | *Vitvi01g01642\|Vitvi01g01642_t001* |
| NF-YC | *Vitvi05g00435\|Vitvi05g00435_t001* |
| NZZ/SPL | *Vitvi19g00320\|Vitvi19g00320_t001* |
| S1Fa-like | *Vitvi08g02161\|Vitvi08g02161_t001* |
| S1Fa-like | *Vitvi08g02161\|Vitvi08g02161_t003* |
| TALE | *Vitvi01g00694\|Vitvi01g00694_t001* |
| TALE | *Vitvi08g01274\|Vitvi08g01274_t001* |
| TALE | *Vitvi18g00602\|Vitvi18g00602_t001* |
| TCP | *Vitvi10g01466\|Vitvi10g01466_t001* |
| TCP | *Vitvi14g01398\|Vitvi14g01398_t001* |
| TCP | *Vitvi14g01519\|Vitvi14g01519_t001* |
| Trihelix | *Vitvi17g01050\|Vitvi17g01050_t001* |
| WOX | *Vitvi01g00427\|Vitvi01g00427_t001* |
| WOX | *Vitvi10g00519\|Vitvi10g00519_t001* |
| WRKY | *Vitvi01g00940\|Vitvi01g00940_t001* |
| WRKY | *Vitvi04g00133\|Vitvi04g00133_t001* |
| WRKY | *Vitvi07g00434\|Vitvi07g00434_t001* |
| WRKY | *Vitvi11g00694\|Vitvi11g00694_t001* |
| WRKY | *Vitvi13g00189\|Vitvi13g00189_t001* |
| WRKY | *Vitvi13g01916\|Vitvi13g01916_t001* |
| YABBY | *Vitvi11g00492\|Vitvi11g00492_t001* |
| YABBY | *Vitvi15g00708\|Vitvi15g00708_t001* |
| ZF-HD | *Vitvi14g01956\|Vitvi14g01956_t001* |
| ZF-HD | *Vitvi17g01082\|Vitvi17g01082_t001* |
| *Down-regulated in Ga.6-1-269 (S)* | |
| AP2 | *Vitvi06g00360\|Vitvi06g00360_t001* |
| AP2 | *Vitvi07g01706\|Vitvi07g01706_t001* |
| AP2 | *Vitvi11g01231\|Vitvi11g01231_t001* |
| ARF | *Vitvi07g00238\|Vitvi07g00238_t001* |
| ARF | *Vitvi10g00854\|Vitvi10g00854_t001* |
| B3 | *Vitvi08g01412\|Vitvi08g01412_t001* |
| BES1 | *Vitvi10g01901\|Vitvi10g01901_t001* |
| bHLH | *Vitvi01g00232\|Vitvi01g00232_t001* |
| bHLH | *Vitvi01g00964\|Vitvi01g00964_t001* |
| bHLH | *Vitvi01g01757\|Vitvi01g01757_t001* |
| bHLH | *Vitvi01g01946\|Vitvi01g01946_t001* |
| bHLH | *Vitvi02g00231\|Vitvi02g00231_t001* |
| bHLH | *Vitvi02g00709\|Vitvi02g00709_t001* |
| bHLH | *Vitvi03g00635\|Vitvi03g00635_t001* |
| bHLH | *Vitvi03g00791\|Vitvi03g00791_t001* |
| bHLH | *Vitvi05g00711\|Vitvi05g00711_t002* |
| bHLH | *Vitvi06g01345\|Vitvi06g01345_t001* |
| bHLH | *Vitvi07g01475\|Vitvi07g01475_t001* |
| bHLH | *Vitvi07g01740\|Vitvi07g01740_t001* |
| bHLH | *Vitvi07g02613\|Vitvi07g02613_t001* |
| bHLH | *Vitvi08g01649\|Vitvi08g01649_t001* |
| bHLH | *Vitvi09g00227\|Vitvi09g00227_t001* |
| bHLH | *Vitvi12g00200\|Vitvi12g00200_t001* |
| bHLH | *Vitvi13g01764\|Vitvi13g01764_t001* |
| bHLH | *Vitvi14g00277\|Vitvi14g00277_t001* |
| bHLH | *Vitvi14g01508\|Vitvi14g01508_t001* |
| bHLH | *Vitvi17g00311\|Vitvi17g00311_t001* |
| bHLH | *Vitvi18g00463\|Vitvi18g00463_t001* |
| bHLH | *Vitvi18g00567\|Vitvi18g00567_t001* |
| bHLH | *Vitvi18g00766\|Vitvi18g00766_t001* |
| bZIP | *Vitvi07g00413\|Vitvi07g00413_t001* |
| bZIP | *Vitvi08g01710\|Vitvi08g01710_t001* |
| bZIP | *Vitvi14g00473\|Vitvi14g00473_t001* |
| bZIP | *Vitvi19g02076\|Vitvi19g02076_t001* |
| C2H2 | *Vitvi01g00552\|Vitvi01g00552_t001* |
| C2H2 | *Vitvi06g00195\|Vitvi06g00195_t001* |
| C2H2 | *Vitvi06g01353\|Vitvi06g01353_t001* |
| C2H2 | *Vitvi07g02262\|Vitvi07g02262_t001* |
| C2H2 | *Vitvi13g01748\|Vitvi13g01748_t001* |
| C2H2 | *Vitvi14g02957\|Vitvi14g02957_t001* |
| C2H2 | *Vitvi19g00397\|Vitvi19g00397_t001* |
| C3H | *Vitvi04g00047\|Vitvi04g00047_t001* |
| C3H | *Vitvi07g01864\|Vitvi07g01864_t001* |
| C3H | *Vitvi08g01816\|Vitvi08g01816_t001* |
| C3H | *Vitvi12g00276\|Vitvi12g00276_t001* |
| CO-like | *Vitvi01g00288\|Vitvi01g00288_t001* |
| CO-like | *Vitvi10g00219\|Vitvi10g00219_t001* |
| CO-like | *Vitvi14g01487\|Vitvi14g01487_t001* |
| DBB | *Vitvi03g00026\|Vitvi03g00026_t001* |
| DBB | *Vitvi18g02424\|Vitvi18g02424_t001* |
| Dof | *Vitvi03g00430\|Vitvi03g00430_t001* |
| Dof | *Vitvi08g00110\|Vitvi08g00110_t001* |
| Dof | *Vitvi18g00858\|Vitvi18g00858_t001* |
| Dof | *Vitvi18g01254\|Vitvi18g01254_t001* |
| E2F/DP | *Vitvi08g01388\|Vitvi08g01388_t001* |
| EIL | *Vitvi06g00166\|Vitvi06g00166_t001* |
| ERF | *Vitvi01g01826\|Vitvi01g01826_t001* |
| ERF | *Vitvi04g00479\|Vitvi04g00479_t001* |
| ERF | *Vitvi05g00715\|Vitvi05g00715_t001* |
| ERF | *Vitvi05g01722\|Vitvi05g01722_t001* |
| ERF | *Vitvi05g01723\|Vitvi05g01723_t001* |
| ERF | *Vitvi05g01724\|Vitvi05g01724_t001* |
| ERF | *Vitvi06g01414\|Vitvi06g01414_t001* |
| ERF | *Vitvi07g01874\|Vitvi07g01874_t001* |
| ERF | *Vitvi07g02066\|Vitvi07g02066_t001* |
| ERF | *Vitvi08g01501\|Vitvi08g01501_t001* |
| ERF | *Vitvi08g01965\|Vitvi08g01965_t001* |
| ERF | *Vitvi12g00348\|Vitvi12g00348_t001* |
| ERF | *Vitvi14g01067\|Vitvi14g01067_t001* |
| ERF | *Vitvi14g02001\|Vitvi14g02001_t001* |
| ERF | *Vitvi15g00947\|Vitvi15g00947_t001* |
| ERF | *Vitvi15g01204\|Vitvi15g01204_t001* |
| ERF | *Vitvi17g00025\|Vitvi17g00025_t001* |
| ERF | *Vitvi18g01617\|Vitvi18g01617_t001* |
| G2-like | *Vitvi01g00578\|Vitvi01g00578_t001* |
| GATA | *Vitvi15g00636\|Vitvi15g00636_t001* |
| GRAS | *Vitvi02g00370\|Vitvi02g00370_t001* |
| GRAS | *Vitvi04g01696\|Vitvi04g01696_t001* |
| GRAS | *Vitvi06g00490\|Vitvi06g00490_t001* |
| GRAS | *Vitvi08g04002\|Vitvi08g04002_t001* |
| GRAS | *Vitvi13g01865\|Vitvi13g01865_t001* |
| GRAS | *Vitvi14g04561\|Vitvi14g04561_t001* |
| GRAS | *Vitvi18g00300\|Vitvi18g00300_t001* |
| GRAS | *Vitvi19g01706\|Vitvi19g01706_t001* |
| GRF | *Vitvi02g00449\|Vitvi02g00449_t001* |
| HD-ZIP | *Vitvi02g00772\|Vitvi02g00772_t001* |
| HD-ZIP | *Vitvi02g00772\|Vitvi02g00772_t002* |
| HD-ZIP | *Vitvi04g01244\|Vitvi04g01244_t001* |
| HD-ZIP | *Vitvi10g00913\|Vitvi10g00913_t001* |
| HD-ZIP | *Vitvi12g00253\|Vitvi12g00253_t001* |
| HD-ZIP | *Vitvi15g00839\|Vitvi15g00839_t001* |
| HSF | *Vitvi05g00561\|Vitvi05g00561_t001* |
| HSF | *Vitvi07g00078\|Vitvi07g00078_t001* |
| HSF | *Vitvi07g00078\|Vitvi07g00078_t002* |
| HSF | *Vitvi08g01513\|Vitvi08g01513_t001* |
| HSF | *Vitvi18g00777\|Vitvi18g00777_t001* |
| LBD | *Vitvi01g00290\|Vitvi01g00290_t001* |
| LBD | *Vitvi03g00628\|Vitvi03g00628_t001* |
| LBD | *Vitvi06g00336\|Vitvi06g00336_t001* |
| LBD | *Vitvi06g00772\|Vitvi06g00772_t001* |
| LBD | *Vitvi13g00545\|Vitvi13g00545_t001* |
| LBD | *Vitvi14g01193\|Vitvi14g01193_t001* |
| LBD | *Vitvi15g00736\|Vitvi15g00736_t001* |
| LBD | *Vitvi18g00677\|Vitvi18g00677_t001* |
| LFY | *Vitvi17g00021\|Vitvi17g00021_t001* |
| MIKC_MADS | *Vitvi02g00427\|Vitvi02g00427_t001* |
| MIKC_MADS | *Vitvi18g00221\|Vitvi18g00221_t001* |
| MIKC_MADS | *Vitvi18g00700\|Vitvi18g00700_t001* |
| MYB | *Vitvi01g01271\|Vitvi01g01271_t001* |
| MYB | *Vitvi02g01019\|Vitvi02g01019_t001* |
| MYB | *Vitvi02g01307\|Vitvi02g01307_t001* |
| MYB | *Vitvi02g01823\|Vitvi02g01823_t001* |
| MYB | *Vitvi03g00559\|Vitvi03g00559_t001* |
| MYB | *Vitvi04g00153\|Vitvi04g00153_t001* |
| MYB | *Vitvi05g00861\|Vitvi05g00861_t001* |
| MYB | *Vitvi07g00515\|Vitvi07g00515_t001* |
| MYB | *Vitvi07g02075\|Vitvi07g02075_t001* |
| MYB | *Vitvi07g03055\|Vitvi07g03055_t001* |
| MYB | *Vitvi08g04220\|Vitvi08g04220_t001* |
| MYB | *Vitvi08g04221\|Vitvi08g04221_t001* |
| MYB | *Vitvi11g00320\|Vitvi11g00320_t001* |
| MYB | *Vitvi12g00376\|Vitvi12g00376_t001* |
| MYB | *Vitvi12g00632\|Vitvi12g00632_t001* |
| MYB | *Vitvi13g01266\|Vitvi13g01266_t001* |
| MYB | *Vitvi13g01266\|Vitvi13g01266_t002* |
| MYB | *Vitvi14g01247\|Vitvi14g01247_t001* |
| MYB | *Vitvi16g00098\|Vitvi16g00098_t001* |
| MYB | *Vitvi16g01017\|Vitvi16g01017_t001* |
| MYB | *Vitvi17g00623\|Vitvi17g00623_t001* |
| MYB | *Vitvi19g00306\|Vitvi19g00306_t001* |
| MYB_related | *Vitvi02g00645\|Vitvi02g00645_t001* |
| MYB_related | *Vitvi04g00076\|Vitvi04g00076_t001* |
| MYB_related | *Vitvi06g00488\|Vitvi06g00488_t001* |
| MYB_related | *Vitvi16g00129\|Vitvi16g00129_t001* |
| NAC | *Vitvi01g00999\|Vitvi01g00999_t001* |
| NAC | *Vitvi02g00242\|Vitvi02g00242_t001* |
| NAC | *Vitvi04g00236\|Vitvi04g00236_t001* |
| NAC | *Vitvi04g00837\|Vitvi04g00837_t001* |
| NAC | *Vitvi12g00255\|Vitvi12g00255_t001* |
| NAC | *Vitvi15g00889\|Vitvi15g00889_t001* |
| NAC | *Vitvi18g00052\|Vitvi18g00052_t001* |
| NAC | *Vitvi18g02404\|Vitvi18g02404_t001* |
| NAC | *Vitvi19g01561\|Vitvi19g01561_t001* |
| NF-YB | *Vitvi10g02273\|Vitvi10g02273_t001* |
| Nin-like | *Vitvi04g04452\|Vitvi04g04452_t001* |
| RAV | *Vitvi14g01248\|Vitvi14g01248_t001* |
| SBP | *Vitvi01g01660\|Vitvi01g01660_t002* |
| SBP | *Vitvi01g01660\|Vitvi01g01660_t004* |
| SBP | *Vitvi01g01678\|Vitvi01g01678_t001* |
| SBP | *Vitvi15g00620\|Vitvi15g00620_t001* |
| SBP | *Vitvi15g00620\|Vitvi15g00620_t002* |
| SBP | *Vitvi18g00027\|Vitvi18g00027_t001* |
| TCP | *Vitvi15g00765\|Vitvi15g00765_t001* |
| Trihelix | *Vitvi04g01604\|Vitvi04g01604_t001* |
| Trihelix | *Vitvi08g01540\|Vitvi08g01540_t001* |
| WRKY | *Vitvi01g01680\|Vitvi01g01680_t001* |
| WRKY | *Vitvi10g00732\|Vitvi10g00732_t001* |
| WRKY | *Vitvi12g00388\|Vitvi12g00388_t001* |
| WRKY | *Vitvi15g01087\|Vitvi15g01087_t001* |
| YABBY | *Vitvi02g00510\|Vitvi02g00510_t001* |
| ZF-HD | *Vitvi01g01013\|Vitvi01g01013_t001* |
| ZF-HD | *Vitvi18g00972\|Vitvi18g00972_t001* |
| *Up-regulated in Ga.12-3-22 (W)* | |
| ARR-B | *Vitvi17g01017\|Vitvi17g01017_t001* |
| B3 | *Vitvi07g01762\|Vitvi07g01762_t001* |
| B3 | *Vitvi15g01244\|Vitvi15g01244_t001* |
| BES1 | *Vitvi18g00924\|Vitvi18g00924_t001* |
| bHLH | *Vitvi01g00876\|Vitvi01g00876_t001* |
| bHLH | *Vitvi01g01482\|Vitvi01g01482_t001* |
| bHLH | *Vitvi01g01745\|Vitvi01g01745_t001* |
| bHLH | *Vitvi02g00231\|Vitvi02g00231_t001* |
| bHLH | *Vitvi04g01361\|Vitvi04g01361_t001* |
| bHLH | *Vitvi08g00979\|Vitvi08g00979_t001* |
| bHLH | *Vitvi15g00906\|Vitvi15g00906_t001* |
| bHLH | *Vitvi17g00046\|Vitvi17g00046_t001* |
| bZIP | *Vitvi01g01342\|Vitvi01g01342_t001* |
| bZIP | *Vitvi03g00292\|Vitvi03g00292_t001* |
| C3H | *Vitvi12g00449\|Vitvi12g00449_t001* |
| CO-like | *Vitvi17g00328\|Vitvi17g00328_t001* |
| Dof | *Vitvi01g01026\|Vitvi01g01026_t001* |
| Dof | *Vitvi15g00936\|Vitvi15g00936_t001* |
| Dof | *Vitvi16g01384\|Vitvi16g01384_t001* |
| ERF | *Vitvi01g00645\|Vitvi01g00645_t001* |
| ERF | *Vitvi04g00190\|Vitvi04g00190_t001* |
| ERF | *Vitvi05g00715\|Vitvi05g00715_t001* |
| ERF | *Vitvi05g01722\|Vitvi05g01722_t001* |
| ERF | *Vitvi06g00052\|Vitvi06g00052_t001* |
| ERF | *Vitvi07g00590\|Vitvi07g00590_t001* |
| ERF | *Vitvi08g01501\|Vitvi08g01501_t001* |
| ERF | *Vitvi10g00522\|Vitvi10g00522_t001* |
| ERF | *Vitvi13g00080\|Vitvi13g00080_t001* |
| ERF | *Vitvi14g00564\|Vitvi14g00564_t001* |
| ERF | *Vitvi14g02001\|Vitvi14g02001_t001* |
| ERF | *Vitvi15g00947\|Vitvi15g00947_t001* |
| ERF | *Vitvi16g00350\|Vitvi16g00350_t001* |
| FAR1 | *Vitvi05g01499\|Vitvi05g01499_t001* |
| FAR1 | *Vitvi07g02480\|Vitvi07g02480_t001* |
| FAR1 | *Vitvi07g02480\|Vitvi07g02480_t002* |
| FAR1 | *Vitvi10g00230\|Vitvi10g00230_t001* |
| FAR1 | *Vitvi18g00039\|Vitvi18g00039_t001* |
| G2-like | *Vitvi02g01004\|Vitvi02g01004_t001* |
| G2-like | *Vitvi08g01723\|Vitvi08g01723_t001* |
| G2-like | *Vitvi08g01834\|Vitvi08g01834_t001* |
| G2-like | *Vitvi08g01834\|Vitvi08g01834_t002* |
| G2-like | *Vitvi19g01742\|Vitvi19g01742_t001* |
| GATA | *Vitvi09g00311\|Vitvi09g00311_t001* |
| GeBP | *Vitvi19g01979\|Vitvi19g01979_t001* |
| GRAS | *Vitvi06g00490\|Vitvi06g00490_t001* |
| GRAS | *Vitvi07g00418\|Vitvi07g00418_t001* |
| GRAS | *Vitvi13g01556\|Vitvi13g01556_t001* |
| GRAS | *Vitvi13g01556\|Vitvi13g01556_t002* |
| GRAS | *Vitvi14g04561\|Vitvi14g04561_t001* |
| HD-ZIP | *Vitvi14g01786\|Vitvi14g01786_t001* |
| HD-ZIP | *Vitvi18g00603\|Vitvi18g00603_t001* |
| HSF | *Vitvi05g00561\|Vitvi05g00561_t001* |
| HSF | *Vitvi07g00078\|Vitvi07g00078_t001* |
| HSF | *Vitvi07g00078\|Vitvi07g00078_t002* |
| HSF | *Vitvi07g01749\|Vitvi07g01749_t001* |
| LBD | *Vitvi01g00291\|Vitvi01g00291_t001* |
| LBD | *Vitvi13g00109\|Vitvi13g00109_t001* |
| LBD | *Vitvi14g01193\|Vitvi14g01193_t001* |
| LBD | *Vitvi19g01589\|Vitvi19g01589_t001* |
| MIKC_MADS | *Vitvi15g00774\|Vitvi15g00774_t001* |
| MIKC_MADS | *Vitvi15g00774\|Vitvi15g00774_t003* |
| MIKC_MADS | *Vitvi15g00776\|Vitvi15g00776_t001* |
| MIKC_MADS | *Vitvi18g01044\|Vitvi18g01044_t001* |
| M-type_MADS | *Vitvi00g04808\|Vitvi00g04808_t001* |
| M-type_MADS | *Vitvi07g02071\|Vitvi07g02071_t001* |
| MYB | *Vitvi01g00302\|Vitvi01g00302_t001* |
| MYB | *Vitvi01g01028\|Vitvi01g01028_t001* |
| MYB | *Vitvi02g01019\|Vitvi02g01019_t001* |
| MYB | *Vitvi02g01308\|Vitvi02g01308_t001* |
| MYB | *Vitvi03g00559\|Vitvi03g00559_t001* |
| MYB | *Vitvi04g00158\|Vitvi04g00158_t001* |
| MYB | *Vitvi08g00069\|Vitvi08g00069_t001* |
| MYB | *Vitvi10g00345\|Vitvi10g00345_t001* |
| MYB | *Vitvi11g00097\|Vitvi11g00097_t001* |
| MYB | *Vitvi14g00974\|Vitvi14g00974_t001* |
| MYB | *Vitvi15g04655\|Vitvi15g04655_t001* |
| MYB | *Vitvi16g00098\|Vitvi16g00098_t001* |
| MYB | *Vitvi16g00125\|Vitvi16g00125_t001* |
| MYB | *Vitvi16g01015\|Vitvi16g01015_t001* |
| MYB | *Vitvi17g00159\|Vitvi17g00159_t001* |
| MYB | *Vitvi17g00231\|Vitvi17g00231_t001* |
| MYB | *Vitvi19g00508\|Vitvi19g00508_t001* |
| MYB | *Vitvi19g01749\|Vitvi19g01749_t001* |
| MYB_related | *Vitvi01g00867\|Vitvi01g00867_t001* |
| MYB_related | *Vitvi04g00076\|Vitvi04g00076_t001* |
| MYB_related | *Vitvi11g00197\|Vitvi11g00197_t001* |
| MYB_related | *Vitvi18g00973\|Vitvi18g00973_t001* |
| NAC | *Vitvi04g00837\|Vitvi04g00837_t001* |
| NAC | *Vitvi12g00255\|Vitvi12g00255_t001* |
| NAC | *Vitvi12g01655\|Vitvi12g01655_t001* |
| NF-YB | *Vitvi05g00299\|Vitvi05g00299_t001* |
| NF-YB | *Vitvi06g01492\|Vitvi06g01492_t001* |
| NF-YB | *Vitvi10g02273\|Vitvi10g02273_t001* |
| Nin-like | *Vitvi04g04452\|Vitvi04g04452_t001* |
| Trihelix | *Vitvi17g01050\|Vitvi17g01050_t001* |
| WRKY | *Vitvi04g00596\|Vitvi04g00596_t001* |
| WRKY | *Vitvi07g00434\|Vitvi07g00434_t001* |
| ZF-HD | *Vitvi12g02135\|Vitvi12g02135_t001* |
| *Down-regulated in Ga.12-3-22 (W)* | |
| bHLH | *Vitvi07g01475\|Vitvi07g01475_t001* |
| ERF | *Vitvi18g02191\|Vitvi18g02191_t001* |
| ERF | *Vitvi02g04095\|Vitvi02g04095_t001* |
| G2-like | *Vitvi01g00578\|Vitvi01g00578_t001* |
| G2-like | *Vitvi01g00249\|Vitvi01g00249_t001* |
| HD-ZIP | *Vitvi10g01773\|Vitvi10g01773_t001* |
| HD-ZIP | *Vitvi08g01752\|Vitvi08g01752_t001* |
| HSF | *Vitvi08g01513\|Vitvi08g01513_t001* |
| LBD | *Vitvi07g01610\|Vitvi07g01610_t001* |
| WRKY | *Vitvi17g00556\|Vitvi17g00556_t001* |
| WRKY | *Vitvi18g00742\|Vitvi18g00742_t001* |
| WRKY | *Vitvi07g01694\|Vitvi07g01694_t001* |

**Table S5.** Genes encoding transcription factors from the top three represented transcription factor families: bHLH, MYB, ERF. Only genes with a Log2FC ≥ 2 and a p-value ≤ 0.05 are shown.

| **Ensembl Gene ID** | **Putative ATH Orthologue** | **NCBI Protein ID** | **Ga. 6-1-269 (S)** | **Ga. 12-3-22 (W)** | **Gene Annotation** |
| --- | --- | --- | --- | --- | --- |
|  |  |  | **Fold change (log2)** | **Fold change (log2)** |  |
| *bHLH Family* |  |  |  |  |  |
| *VIT_01S0026G00010* | *AT1G10586.1* | XP_019079355.1 | -7.22 | - | transcription factor bHLH168-like isoform X1 |
| *VIT_17S0000G03550* | *AT5G65640.1* | XP_002276557.3 | -7.16 | - | transcription factor bHLH93 |
| *VIT_01S0011G03720* | *AT5G65640.1* | XP_002279412.1 | -6.07 | - | transcription factor bHLH93 |
| *No ensembl gene ID* | *AT1G10586.1* | XP_019079355.1 | -4.91 | - | transcription factor bHLH75-like |
| *VIT_07S0205G00180* | *AT4G37850.1* | XP_010652695.1 | -4.89 | - | transcription factor bHLH168-like isoform X1 |
| *VIT_00S0479G00020* | *AT4G20970.1* | XP_003635365.2 | -3.73 | - | transcription factor bHLH18-like isoform X1 |
| *VIT_13S0064G01290* | *AT2G42280.3* | XP_003633592.1 | -3.64 | - | transcription factor bHLH162 |
| *VIT_00S0927G00010* | *AT4G37850.1* | XP_002276304.3 | -3.06 | - | transcription factor bHLH130 |
| *VIT_13S0019G00540* | *AT2G31730.1* | XP_002279297.2 | -2.94 | - | transcription factor bHLH18 isoform X2 |
| *VIT_00S1314G00010* | *AT4G37850.1* | XP_010647474.1 | -2.92 | - | transcription factor bHLH123 isoform X1 |
| *VIT_01S0011G02940* | *AT1G68810.1* | XP_010647981.1 | -2.51 | - | transcription factor bHLH18 |
| *VIT_18S0001G06650* | *AT1G72210.1* | XP_002285733.2 | -2.35 | - | transcription factor bHLH30 isoform X1 |
| *VIT_02S0012G01450* | *AT5G46690.1* | XP_002278824.1 | -2.35 | - | transcription factor bHLH96 |
| *VIT_18S0001G10300* | *AT1G49770.1* | XP_002284878.3 | -2.32 | - | transcription factor bHLH71-like |
| *VIT_14S0068G01580* | *AT1G68810.1* | XP_002274955.1 | -2.07 | - | transcription factor bHLH95-like |
| *VIT_14S0128G00110* | *AT1G72210.1* | XP_002281083.2 | -2.06 | - | transcription factor bHLH30 |
| *VIT_08S0007G07810* | *AT2G42280.1* | XP_002277274.1 | -2.02 | - | transcription factor bHLH94 |
| *VIT_05S0094G00480* | *AT2G31730.1* | XP_002273925.1 | 2.31 | - | transcription factor bHLH130 |
| *VIT_11S0016G02070* | *AT5G56960.1* | XP_002279486.2 | 2.8 | - | transcription factor bHLH113 |
| *VIT_17S0000G08150* | *AT4G20970.1* | XP_002279841.1 | 2.86 | - | putative transcription factor bHLH041 |
| *VIT_01S0010G02070* | *AT1G31050.1* | XP_002268359.2 | 4.19 | - | transcription factor bHLH162-like isoform X1 |
| *VIT_14S0081G00720* | *AT4G14410.1* | XP_002268590.2 | 4.54 | - | transcription factor bHLH111 |
| *VIT_01S0244G00010* | *AT5G50915.2* | XP_002267465.1 | - | 2.05 | transcription factor bHLH104 |
| *VIT_07S0191G00240* | *AT1G72210.1* | XP_010652690.1 | - | -7.61 | transcription factor bHLH94-like |
| *MYB Family* |  |  |  |  |  |
| *VIT_02S0025G00320* | *AT1G63910.1* | XP_002273004.1 | -6.01 | - | transcription factor MYB86 |
| *VIT_12S0134G00570* | *AT4G21440.1* | XP_002272561.1 | -5.89 | - | transcription factor MYB39 |
| *VIT_06S0004G02110* | *AT3G08500.1* | XP_002282821.1 | -4.93 | - | transcription factor MYB46 |
| *VIT_04S0008G01830* | *AT4G34990.1* | XP_002279910.2 | -3.87 | - | transcription factor MYB1-like |
| *VIT_19S0014G03820* | *AT4G21440.1* | XP_002281638.1 | -2.99 | - | transcription factor MYB35 |
| *VIT_18S0001G05670* | *AT1G34670.1* | XP_002284142.1 | -2.58 | - | transcription factor MYB93 |
| *VIT_17S0000G04130* | *AT1G49010.1* | XP_002274167.1 | 2.04 | - | transcription factor MYBS1 |
| *VIT_09S0002G01400* | *AT2G16720.1* | XP_002275148.1 | 2.29 | - | transcription factor MYB1-like |
| *VIT_15S0046G00170* | *AT4G05100.1* | NP_001268160.1 | 2.42 | - | MYBPA1 protein |
| *VIT_07S0005G01950* | *AT2G47190.1* | NP_001267917.1 | 2.7 | - | transcription factor MYB108-like |
| *VIT_02S0033G00300* | *AT5G45580.2* | XP_002264257.2 | 2.7 | 4.37 | myb family transcription factor PHL11 |
| *VIT_10S0116G00500* | *AT2G46410.1* | XP_002267669.1 | 2.94 | - | MYB-like transcription factor ETC1 |
| *VIT_19S0085G00890* | *AT1G79430.2* | XP_002265800.3 | 3.63 | - | myb family transcription factor APL isoform X2 |
| *VIT_06S0004G04140* | *AT5G59780.3* | XP_019076057.1 | 3.73 | - | transcription factor MYB59 |
| *VIT_08S0007G07580* | *AT3G04030.3* | XP_010651406.1 | 3.75 | - | myb-related protein 2 isoform X3 |
| *No ensembl gene ID* | *AT3G04030.3* | XP_010653833.1 | 4.08 | 2.07 | myb-related protein 2-like |
| *VIT_08S0007G06310* | *AT5G06800.2* | XP_002278675.1 | 4.75 | - | myb family transcription factor PHL5 isoform X2 |
| *VIT_00S0203G00170* | *AT1G22640.1* | XP_019073658.1 | 7.8 | - | transcription factor MYB4-like isoform X1 |
| *VIT_15S0048G02120* | *AT5G02320.2* | XP_019081315.1 | - | 4.96 | transcriptional activator Myb |
| *VIT_01S0011G03730* | *AT1G68320.1* | XP_002279433.1 | - | 8.46 | transcription factor MYB62-like |
| *VIT_18S0001G15260* | *AT1G17950.1* | XP_002278632.2 | - | -7.15 | transcription factor MYB73-like |
| *ETHYLENE RESPONSE FACTORS* | | | | | |
| *VIT_14S0081G00730* | *AT3G23240.1* | XP_010660112.1 | 7.05 | - | ethylene-response factor C3-like |
| *VIT_17S0000G04480* | *AT5G19790.1* | XP_010663517.1 | 6.4 | - | ethylene-responsive transcription factor RAP2-11-like |
| *VIT_16S0013G01070* | *AT5G51190.1* | XP_002282167.1 | 5.31 | - | ethylene-responsive transcription factor ERF105 |
| *No ensembl gene ID* | *AT5G51190.1* | XP_002281931.1 | 4.06 | - | ethylene-responsive transcription factor ERF105 |
| *VIT_16S0013G01110* | *AT5G51190.1* | XP_002282239.1 | 3.6 | - | ethylene-responsive transcription factor ERF105-like |
| *VIT_16S0013G00950* | *AT5G51190.1* | XP_002281813.1 | 3.53 | - | ethylene-responsive transcription factor ERF105 |
| *VIT_11S0016G00660* | *AT1G19210.1* | XP_002276572.2 | 3.1 | - | ethylene-responsive transcription factor ERF017 |
| *VIT_16S0013G01050* | *AT5G67190.1* | XP_002281966.1 | 3 | - | ethylene-responsive transcription factor 5 |
| *VIT_16S0013G00990* | *AT5G51190.1* | XP_002281947.1 | 2.6 | - | ethylene-responsive transcription factor 5-like |
| *VIT_05S0049G00490* | *AT3G23230.1* | XP_002284760.3 | -2.31 | - | ethylene-responsive transcription factor ERF095 |
| *VIT_05S0077G01860* | *AT3G16770.1* | XP_002272426.1 | -2.31 | - | ethylene-responsive transcription factor RAP2-3 |
| *VIT_17S0000G00200* | *AT5G61890.1* | XP_010663806.2 | -3.28 | - | "ethylene-responsive transcription factor ERF114 |
| *VIT_11S0037G00870* | *AT1G16060.1* | XP_010656633.1 | -3.45 | - | AP2-like ethylene-responsive transcription factor At1g16060 |
| *VIT_11S0052G00840* | *AT1G16060.1* | XP_002268683.1 | -3.58 | - | AP2-like ethylene-responsive transcription factor AIL5 |
| *VIT_05S0049G00500* | *AT3G23230.1* | XP_010650069.1 | -4.49 | - | ethylene-responsive transcription factor ERF098-like |
| *VIT_01S0026G01690* | *AT3G54320.1* | XP_010652396.1 | -5.39 | - | AP2-like ethylene-responsive transcription factor At1g16060 isoform X2 |
| *VIT_09S0002G06750* | *AT1G15360.1* | XP_002268413.2 | -6.24 | - | ethylene-responsive transcription factor WIN1 |
| *VIT_13S0047G00340* | *AT3G54320.1* | XP_010659009.1 | -6.52 | - | ethylene-responsive transcription factor WRI1 |
| *VIT_11S0016G05340* | *AT1G15360.1* | XP_019078421.1 | -7.14 | - | ethylene-responsive transcription factor WIN1 isoform X2 |
| *VIT_02S0025G04440* | *AT5G52020.1* | XP_002276184.1 | - | -3.25 | ethylene-responsive transcription factor ERF026 |
| *VIT_18S0001G10150* | *AT5G51190.1* | XP_002276994.2 | - | -2.69 | ethylene-responsive transcription factor ERF020 |

**Table S6.** List of genes and corresponding primers used for RT-qPCR validation of RNA-sequencing data. NS indicates that a gene was not significantly detected by RNA-sequencing. Internal control gene, *VvActin,* is listed at the bottom.

| **Gene ID** | **Log2FC Ga. 6-1-269 (S)** | **Log2FC Ga. 12-3-22 (W)** | **Gene Definition** | **Forward Primer** | | **Reverse primer** |
| --- | --- | --- | --- | --- | --- | --- |
| *Oppositely regulated* | | | | | | |
| *VIT_18S0001G12900  (VvJMT)* | -4.12 | 2.02 | jasmonate O-methyltransferase | | CCACTTCGTGCACTCTTCTT | CGCAATCTGGACTCGTCTTT |
| *VIT_10S0003G05420  (VvBBE15)* | -3.95 | 2.14 | berberine bridge enzyme-like 15 | | CCCGATGTAAAGCACTGATCT | GTGGACAGACTCCTCCAAATAAT |
| *VIT_15S0021G00330  (VvNPF7.3)* | -2.45 | 3.43 | protein NRT1/ PTR FAMILY 7.3 | | GGGAAATTGTCGCAGAAAGATG | GACTCTCCTTGTCTTCCTGTTC |
| *VIT_00s0779g00010 (VvUnk)* | -1.59 | 1.25 | uncharcterized protein | | CTCGTATCTCTTGGTGGATGTG | CATGCTCCCTCAGACCTAAAC |
| *VIT_02S0012G00530  (VvRPPK1)* | -2.82 | 1.03 | ribose-phosphate pyrophosphokinase 1 isoform X1 | | GGCAGTCCATGGGTTACTTT | CAGGTGAGACCACTACCAAATC |
| *VIT_01S0026G01490  (VvNPF2.13)* | 9.72 | -4.09 | protein NRT1/ PTR FAMILY 2.13 | | GATTGGTCGAACGAGAGAGAAG | GCTTCAGCAAAGCCCATTAG |
| *VIT_00S0317G00110  (VvHIPP7)* | 1.34 | -2.91 | heavy metal-associated isoprenylated plant protein 7 | | GTGAGCCGCCTGAGAATATAG | TCCTTCTCCAACCTTGAGTTTC |
| *VIT_08S0007G01050  (VvAKR)* | 1.12 | -1.53 | "NADPH-dependent aldo-keto reductase | | CCTTCCCAAGAGTGTACATGAG | AAGCCTTTGCTGGTGGATAG |
| *VIT_12S0028G03270  (VvERF4)* | 1.47 | -1.68 | ethylene-responsive transcription factor 4 | | GTGGCTTTCCGTTCCATTAC | CCGACAAACGCATCCAAA |
| *VIT_01S0011G03110  (VvMYB-EFM)* | 1.85 | -1.97 | myb family transcription factor EFM | | GAGGACACACCCAAGAAGATAC | GTTTCAGCCGTAGAACTGGT |
| *Up-regulated in Ga. 6-1-269 (S)* | | | | | | |
| *VIT_05s0049g00280 (VvCRK2)* | 6.17 | NS | cysteine-rich receptor-like protein kinase 2 isoform X2 | | CGCCTTTCTTTGCCGATAAC | CCAGCCACTCCTCCAATAAT |
| *VIT_00s0270g00130 (VvACO1)* | 5.25 | NS | 1-aminocyclopropane-1-carboxylate oxidase homolog 1-like | | CGCCTTTCTTTGCCGATAAC | CCAGCCACTCCTCCAATAAT |
| *VIT_04S0008G05750 (VvWRKY18)* | 9.73 | NS | WRKY transcription factor 18-like | | GCAGAGATGCATGGAGGATAG | GGGCTATCTGGTGAATATGAGG |
| *VIT_04S0023G00320 (VvPIN5)* | 10.12 | NS | auxin efflux carrier component 5 | | CTGGCTCACACTTCTGCTATT | TCCAGGCTCAACCTCTATCA |
| *VIT_13S0019G05260 (Vv4CL5)* | 6.7 | NS | 4-coumarate--CoA ligase-like 5 | | CTTCTGAGTCATCCCAGTGTTC | TCCACCTGCTCTCACTACATA |
| *Up-regulated in Ga. 12-3-22 (W)* | | | | | | |
| *VIT_01s0011g03730 (VvMYB62)* | NS | 8.46 | transcription factor MYB62-like | | CGAGTCTGATAGCAAGAGGTTC | AGCAGCAGTTGAAGAAGTAGAG |
| *VIT_18S0001G14910 (VvCAD6)* | NS | 7.72 | probable cinnamyl alcohol dehydrogenase 6 | | GCCGTCTTTCCCTCTGATATTC | TTCCACACACATCCATCATCTC |
| *VIT_16s0022g00310 (VvRLP-EIX2)* | NS | 7.18 | receptor-like protein EIX2 | | CTGGAGTGGCTTTCTCATCTT | GTGAACGAGAGAAGGGAGTTTAT |
| *VIT_04s0008g00120 (VvCHI)* | NS | 6.68 | basic endochitinase-like | | GGGCTGTGTTGTAGCCAATA | TTGGAGTGGAGGGAGTAGAA |
| *VIT_15s0048g02120 (VvMYB)* | NS | 4.96 | transcriptional activator Myb | | GACCCACATATCAAGGCTAACA | AGAATGAGGTAGTGGAGGAGAA |
| *VvActin* | - | - | - | | GATTTGGTGATCGTGTGAGT | GACARTTTCOCGTTCAGCAGT |

**Table S7**. Genes selected for Arabidopsis loss-of-function mutant analysis and their Arabidopsis Biological Research Center (ABRC) identifier. Log2 fold change is shown for both genotypes, entries with “NS” indicate that expression was not significantly detected through RNA-seq.

| **Gene ID** | **Gene Definition** | **Arabidopsis Ortholog** | | **ABRC Identifier** | **Ga.12-3-22 (W) Log_2_FC** | **Ga.6-1-269 (S) Log_2_FC** |
| --- | --- | --- | --- | --- | --- | --- |
| **(*Vitis vinifera*)** | **(*Vitis vinifera*)** |  |  |  |  |  |
| *VIT_04S0023G00320 (VvPIN5)* | auxin efflux carrier component 5 | | *AT5G16530* | SALK_021738C | NS | 10.1 |
|  |  | |  |  |  |  |
| *VIT_04S0008G05750 (VvWRKY18)* | WRKY transcription factor 18-like | | *AT2G25000* | SALK_120706C |  |  |
|  |  |  |  |  | NS | 9.7 |
| *VIT_01S0011G03730 (VvMYB62)* | transcription factor MYB62-like | | *AT1G68320* | SALK_104944 |  |  |
|  |  |  |  |  | 8.45 | NS |
| *VIT_18S0001G14910 (VvCAD6)* | probable cinnamyl alcohol dehydrogenase 6 | | *AT4G39330* | SALK_081375C |  |  |
|  |  |  |  |  |  | NS |
|  |  |  |  |  | 7.7 |  |
|  |  |  |  |  |  |  |
|  |  |  |  |  |  |  |
| *VIT_13S0019G05260 (Vv4CL5)* | 4-coumarate--CoA ligase-like 5 | | *AT1G20480* | SALK_027694C |  |  |
|  |  |  |  |  | NS | 6.7 |
